# Process Window for Seeded Growth of Arrays of Quasi-Spherical Substrate-Supported Au Nanoparticles

**DOI:** 10.1021/acs.langmuir.1c00693

**Published:** 2021-05-03

**Authors:** Björn Landeke-Wilsmark, Leif Nyholm, Carl Hägglund

**Affiliations:** † Division of Solar Cell Technology, Department of Materials Science and Engineering, Uppsala University, Box 35, 751 03 Uppsala, Sweden; ‡ Department of Chemistry - Ångström Laboratory, Uppsala University, Box 523, 751 20 Uppsala, Sweden

## Abstract

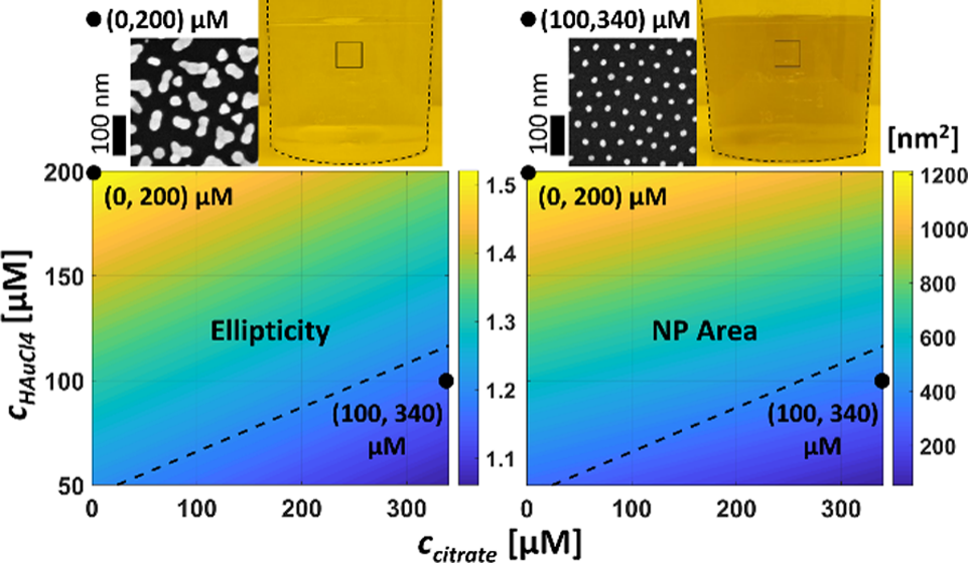

The controlled growth of surface-supported metal nanoparticles (NPs) is essential to a broad range of applications. To this end, we explore the seeded growth of highly ordered arrays of substrate-supported Au NPs through a fully orthogonal design of experiment (DoE) scheme applied to a reaction system consisting of HAuCl_4_, citrate, and hydrogen peroxide. Scanning electron microscopy in combination with digital image analysis (DIA) is used to quantitatively characterize the resultant NP populations in terms of both particle and array features. The effective optical properties of the NP arrays are additionally analyzed using spectroscopic ellipsometry (SE), allowing characteristics of the localized surface plasmon resonances (LSPRs) of the arrays to be quantified. We study the dependence of the DIA- and SE-extracted features on the different reagent concentrations through modeling using multiple linear regression with backward elimination of independent variables. A process window is identified for which uniform arrays of quasi-spherical Au NPs are grown over large surface areas. Aside from reagent concentrations the system is highly sensitive to the hydrodynamic conditions during the deposition. This issue is likely caused by an Au precursor mass-transport limitation of the reduction reaction and it is found that agitation of the growth medium is best avoided to ensure a macroscopically even deposition. Parasitic homogeneous nucleation can also be a challenge and was separately studied in a full DoE scheme with equivalent growth media but without substrates, using optical tracking of the solutions over time. Conditions yielding quasi-spherical surface-supported NPs are found to also be affiliated with strong tendencies for parasitic homogeneous nucleation and thereby loss of Au precursor, but addition of polyvinyl alcohol can possibly help alleviate this issue.

## 1 Introduction

Nanoparticles (NPs) composed of free-electronlike metals, such as Ag, Au, and Al, are of great scientific and technological interest. For example, their ability to support localized surface plasmon resonances (LSPRs) with strong near-field enhancement and generation of hot carriers stimulates interest in a wide range of diverse disciplines including photocatalysis,^1,2^ biosensors,^3^ surface enhanced Raman spectroscopy,^2,4^ and photovoltaics.^5−7^ These applications depend sensitively on the shape, size, and dielectric environment of the particles. The ability to effectively tailor these properties is therefore critical and it is often desirable to do this for NPs in surface-supported large-area arrays.

There are multiple fabrication techniques for implementing such arrays including direct-write methods like electron beam lithography^8^ or nanoimprint lithography,^9^ various colloidal self-assembly schemes,^10,11^ micro-/nanocontact printing,^12^ block copolymer (BCP) lithography,^13−16^ and BCP micellar lithography.^17−19^ Here, we utilize BCP lithography as it offers a facile and inexpensive way for rapid and parallel patterning of large surfaces with highly ordered arrays of nonclose-packed Au NPs of uniform size and shape, thus making it an attractive option for implementing these structures in a scalable way. However, the referenced version of BCP lithography has two drawbacks in this context: first, when using a simple (and thus low-cost) polymer chain geometry, the particle size and interparticle distance cannot readily be decoupled to any great extent. Second, due to practical limitations, the generated particles tend to be fairly small (diameter ≤10 nm). Fortunately, both of these issues can be resolved by introducing a subsequent seeded growth step, which entails the selective deposition of additional metal on pre-existing seed particles.

Seeded growth of substrate-supported NPs comes, however, with its own set of challenges. These include (i) avoiding seed particle desorption in the case of weak particle–substrate adhesion, (ii) achieving growth selectivity between surfaces of different materials, (iii) avoiding parasitic homogeneous nucleation (HN) in the growth medium, and (iv) if necessary, ensuring uniform hydrodynamic conditions over the sample surface during growth. As previously demonstrated, the use of atomic layer deposition (ALD) post-seed formation can be used to satisfactorily address (i). Performing as little as a single ALD cycle of HfO_2_ was proven to be highly effective in preventing particle desorption without hampering subsequent metal deposition.^20^

**Figure 1:**
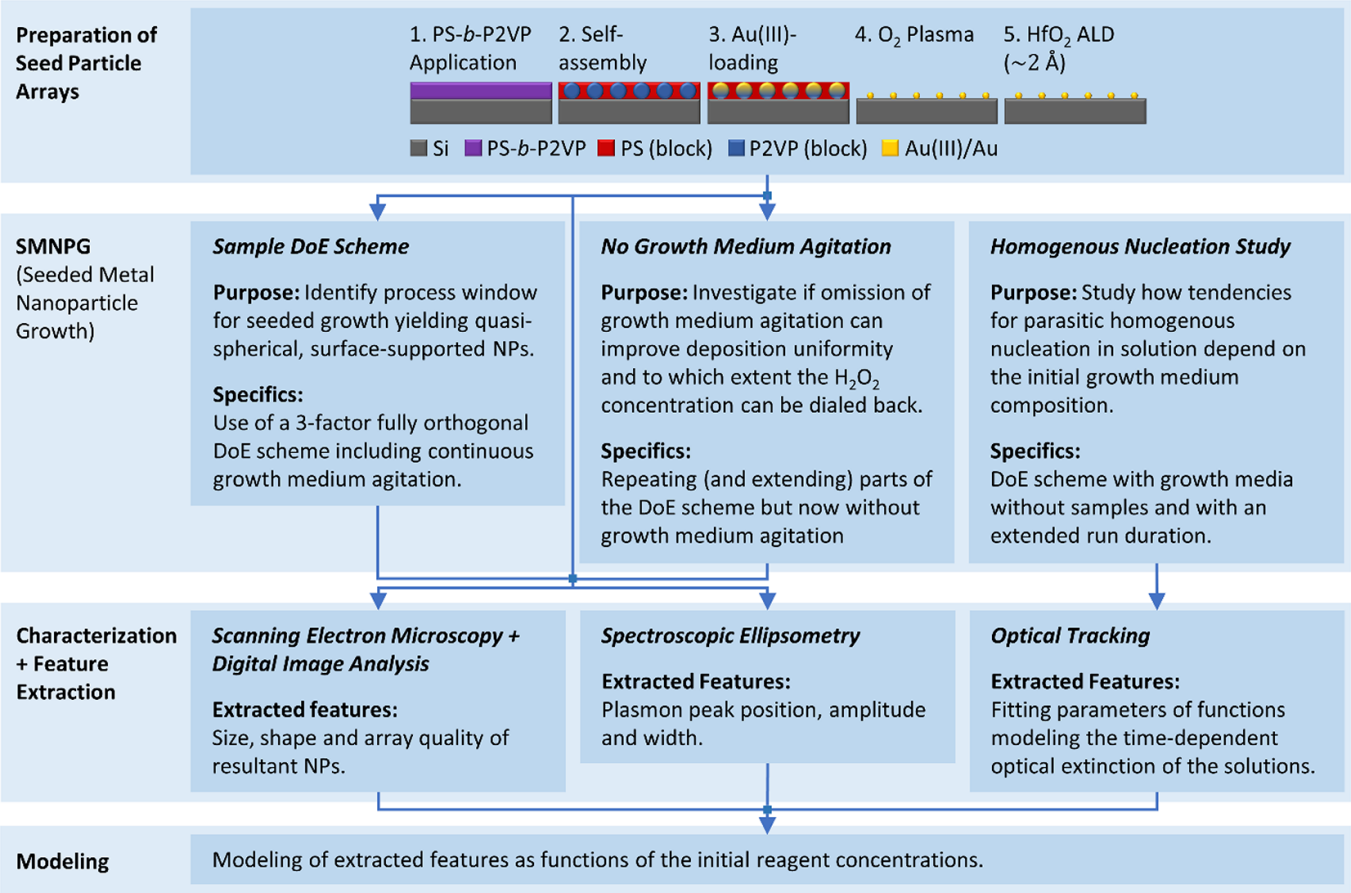
Overview of the experimental work and analysis included in this study. Blue arrows signify the direction of different process flows.

The concept of selective, electroless deposition of Au or Ag on BCP-derived arrays of surface-supported Au seed particles, with the aim of enlarging the Au seeds^21−25^ or implementing core–shell nanostructures,^15,21,26,27^ has been demonstrated previously. In the studies concerning Au, Au(III) was reduced to Au(0) using either hydroxylammonium chloride^21,22^ or a photochemical process involving UV light^23−25^ and less traditional reducing agents. As for the deposition of Ag, either Tollens’ reagent^26^ or hydroquinone^15^ was used. The seeded growth of surface-supported arrays of Au@PNIPAM core-shell NPs^11^ and arrangements of multiple Au NPs inside larger polymer domains^28,29^ has also been demonstrated using a combination of ascorbic acid and CTAB as a weak reducing agent and a surfactant, respectively. A lot of insights and inspiration may, however, be gained by widening the frame of reference to include the extensively explored field of seeded growth of colloidal NPs suspended in solution. Two protocols^30,31^ in particular caught our attention due to their (i) demonstrated ability to generate monodisperse, quasi-spherical NPs with tailored sizes within a large range (17–325 nm in diameter), (ii) rapid one-step procedure conducted at room temperature (RT), and (iii) use of relatively cheap and benign reagents. The adaptation of the growth protocol developed by Liu et al.,^30^ using HAuCl_4_, trisodium citrate (“citrate”), and H_2_O_2_, has previously been investigated by us for use on arrays of surface-supported Au seed particles with encouraging results.^20^ This work is here advanced with a more rigorous, in-depth exploration of the reagent concentration parameter space coupled with quantification of various individual and collective NP features using scanning electron microscopy (SEM), digital image analysis (DIA), and spectroscopic ellipsometry (SE). This further allows us to model these features as functions of the growth medium composition and to identify a reagent concentration process window in which uniform, quasi-spherical NPs can be obtained. Moreover, we also study the effects of growth medium agitation, condition-dependent tendencies for parasitic HN, and how the addition of poly(vinyl alcohol) (PVA) might be used to delay this and improve Au precursor utilization. An overview of the present study is presented in Figure 1.

## 2 Experimental Section

### 2.1 Materials

Poly(styrene-*block*-2-vinylpyridine) (PS-*b*-P2VP, *M_n_* = 44.0-*b*-18.5 kg·mol^–1^, PDI = 1.07) and *homo*polystyrene (*h*PS, *M_n_* = 12.5 kg·mol^–1^, PDI = 1.04) were purchased from Polymer Source Inc., Canada. PS-*b*-P2VP and *h*PS were dissolved in toluene (Selectipur, Merck), while acetone (GPR Rectapur, VWR) and 2-propanol (IPA, GPR Rectapur, VWR) were used for sample cleaning. Hydrogen tetrachloroaurate(III) trihydrate (HAuCl_4_ · 3H_2_O, ACS reagent, ≥49.0% Au basis, Fluka), trisodium citrate dihydrate (Na_3_C_6_H_5_O_7_ · 2H_2_O, ≥99%, Alfa Aesar), hydrogen peroxide (H_2_O_2_, 31%, VLSI Selectipur, BASF), PVA (*M_w_*∼31 kg·mol^–1^, Mowiol 4-88, Sigma-Aldrich), deionized (DI) water, and Si(100) substrates (SSP, n^++^) were used in various seeded growth procedures. Aqua regia was prepared using hydrochloric acid (HCl, 36% VLSI Sel., BASF) and nitric acid (HNO_3_, 69% VLSI Selectipur, BASF).

### 2.2 Seed Sample Preparation

Four 100 mm Si(100) wafers were spin-rinsed using acetone and IPA sequentially and then ashed (TePla 300, 5 min, 100 W, 50 sccm N_2_ + 50 sccm O_2_) prior to subjecting them to surface functionalization with hexamethyldisilazane. This entailed *∼*30 min vapor-grafting of the species at 150 ^°^C under rough vacuum. Next, BCP films were applied via spin-coating a 0.9% (w/w) toluene solution at 6000 rpm to achieve a film thickness of approximately 21 nm. The polymer films were composed of 30% (w/w) *h*PS_12.5k_ in addition to the PS_44k_-*b*-P2VP_18.5k_ BCP to actively push the system into a sphere-forming geometry during the subsequent self-assembly step. The wafers were sequentially subjected to solvent vapor annealing (SVA) using toluene vapors in a custom-built, flow-based setup in which the partial pressure of toluene (*p_tol_*) can be rapidly and dynamically controlled. The SVA was conducted at RT (21 ^°^C) for 1 h using a steady-state nominal *p_tol_* of 97% of its vapor pressure (*p*_tol_^*^, i.e., *p_tol_*/*p*_tol_^*^ = 0.97). The wafers were then immersed in a 2.5 mM HAuCl_4_(aq) solution for 15 min to selectively load the P2VP domains with AuCl_4_^–^. Next, the wafers were ashed in a two-step procedure (TePla 300, I: 5 min, 100 W, 50 sccm O_2_ + 50 sccm N_2_; II: 5 min, 1000 W, 50 sccm O_2_). This simultaneously removed the polymers and reduced Au(III) to Au(0), thereby generating highly ordered arrays of small Au NPs located at the lattice points of the previous P2VP domains in the BCP template. One ALD cycle of HfO_2_ (Picosun R-200, 170 ^°^C, 1 cycle: 5 s H_2_O, 10 s N_2_, 0.1 s tetrakis(dimethylamido)hafnium(IV), 10 s N_2_. Termination: 0.1 s H_2_O, 10 s N_2_, 5 s H_2_O) was performed on all wafers, to augment the particle–substrate adhesion,^20^ prior to applying (spin-coating, 500 rpm) and soft-baking (hotplate, 110 ^°^C, 10 min) a thick layer of S1813 photoresist. The purpose of the S1813 is to serve as a protective coating during the subsequent dicing (Disco DAD 361) of the wafers into 20 × 20 mm^2^ sample pieces. Several smaller edge pieces, in addition to the nine appropriately sized samples, were obtained per wafer. The S1813 was then stripped using acetone, IPA, and additional ashing (TePla 300, 5 min, 50 W, 50 sccm O_2_ + 50 sccm N_2_). Although one ALD cycle of HfO_2_ can boost the particle adhesion sufficiently, a second ALD cycle was lastly performed to immobilize any dislodged NPs in the immediate vicinity of the dicing cuts. Structure verification using SEM was performed after the precursor-loading of the BCP template and after the last ALD step.

### 2.3 Design of Experiment (DoE) Scheme for Seeded Metal Nanoparticle Growth

The procedure for seeded metal nanoparticle growth (SMNPG) was conducted by immersing individual samples, affixed to disposable sample mounts of polystyrene (PS), in freshly prepared growth media in disposable polypropylene (PP) beakers for 5 min. The samples were attached to the PS mounts using a hotglue resin, which is believed to be inert under these conditions. The PP beakers and PS sample mounts were copiously rinsed with IPA and DI water prior to use. Upon process termination, the samples were removed from the solution, thoroughly rinsed with DI water and IPA, and dried with a N_2_ gun. While immersed, the samples were oriented vertically at the radial edge of the beaker and with the NP-decorated surface facing the center of the vessel. As PTFE-clad magnetic stirring bars were used to generate a mild vortex in the growth medium, a directed flow over the decorated surface was achieved. The risk of cross-contamination between procedures was minimized through the use of a new set of disposable labware and aqua regia-cleaned [3:1 HCl (36%):HNO_3_ (69%)] stirring bar for each sample. (*Caution!* Aqua regia solutions are strongly corrosive and oxidizing; adding organics may cause an explosion).

**Figure 2:**
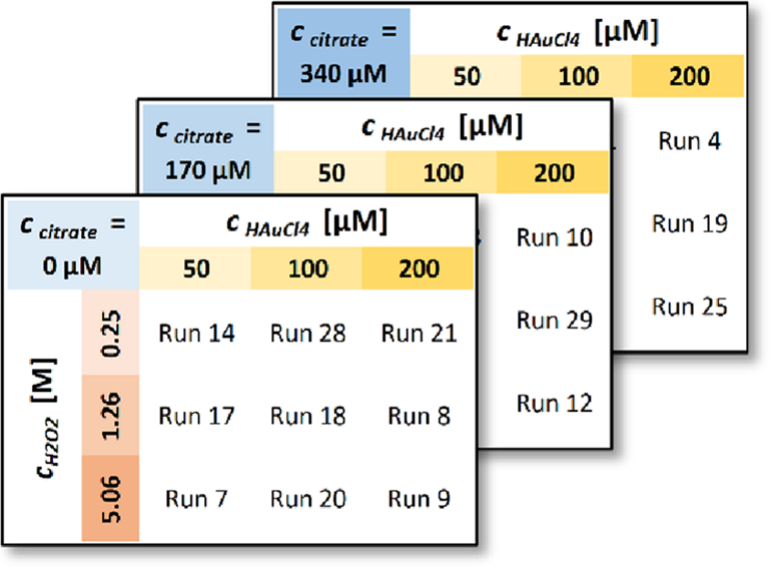
DoE scheme (CP replicates not shown). For clarity, the combinations of *c_citrate_* and *c*_*H*2*O*2_ are the same for all three levels of *c_citrate_*.

To investigate the effects of the reagent concentrations (i.e., *c*_*HAuCl*4_, *c_citrate_*, and *c*_*H*2*O*2_), a fully orthogonal 3-factor DoE scheme, including three centerpoint (CP) replicates, was implemented with levels as shown in Figure 2. The total volume (40 mL) was kept constant and the reagents were added in the following order: DI water, citrate, HAuCl_4_, H_2_O_2_, and lastly the seed-decorated sample. The stock solutions used were 50 mM HAuCl_4_(aq), 1% (w/w) citrate(aq), and 31% (w/w) H_2_O_2_(aq). The DoE runs were all conducted at RT (21 ^°^C) and under yellow light conditions over a span of three consecutive days in a fume hood in a climate-controlled cleanroom environment. To minimize unintentional biasing, the run order and the parent wafer sample origin (i.e., from which one of the four parent wafers of the sample was taken) were randomized. The only constraint imposed regarding the sample origin was that only nine samples could be drawn from any one parent wafer.

### 2.4 DoE Scheme for Growth Media without Samples

To investigate the tendency for HN as a function of growth conditions, the entire DoE scheme was repeated using an extended run duration of 15 min and growth media without immersed samples or sample holders. These runs were conducted over a span of two consecutive days with equipment and experimental conditions otherwise identical to those in the original SMNPG DoE scheme.

### 2.5 Growth without Agitation

In addition to the two DoE schemes, a follow-up experiment was also conducted with the aim to elucidate whether the macroscale deposition uniformity could be improved by omitting the growth medium agitation. The experiment consisted of a series of SMNPG procedures using a progressively decreasing *c*_*H*2*O*2_. The experimental setup was identical to that used in the sample DoE scheme except that instead of using a stirring bar, the reagents were rapidly mixed (upon H_2_O_2_ addition) by drawing the solution once in and out of a 10 mL micropipette prior to sample immersion. The immersion time was also extended from 5 to 10 min to mitigate the effects of a less efficient reagent mass transport.

### 2.6 Characterization of DoE Samples

SEM images were acquired at three specific sample inspection points (IP1–3) using a Zeiss 1530 SEM (*V_acc_*= 5 kV, 0^°^ stage tilt, WD∼3 mm, in-lens SEII detector) post-SMNPG. IP1–3 lie along one of the sample diagonals with IP1 and IP3 closer to the corners, while IP2 is in the sample center (Figure S2b). For reference, the seed particle arrays on two random samples from each parent wafer were inspected prior to the SMNPG.

DIA of one SEM image, acquired at 200 k magnification, was performed for each inspection point of the samples using CellProfiler 3.0 software. In brief, a small sweeping median filter (3 × 3 pixel) was used for initial smoothing and noise reduction prior to object identification using the minimum cross-entropy thresholding algorithm.^32^ Purposefully, no attempt at declumping fused or touching particles was made and NPs located on the image border were excluded from the number count (*N*) and size and shape characterization but included when estimating *ϕ* (defined below). The DIA-extracted parameters include the relative change in NP count (Δ*N*/*N*_0_), where *N*_0_ is obtained from the corresponding wafer reference samples, surface area fraction covered by NPs (*ϕ*), mean values of the NP top-view cross-sectional area (*x̅_Area_*), maximum Ferret diameter (*x̅_Max FD_*), form factor (*x̅_FF_*), and ellipticity (*x̅_Ellips_*) as well as their corresponding SDs (*s_x_*). The form factor (FF) is defined as 4*π* · *area*/*perimeter*^2^ and equals 1 for NPs with perfectly circular top-view cross sections. The ellipticity on the other hand is the ratio of the major to minor axes of an ellipse fitted to the NP cross section. *s_d_* is the SD of the centroid-to-centroid distance between the NPs and their nearest neighbor. *s_θ_* is the SD of the discrepancy between the angle spanned by the centroids of the two nearest neighbors, with the apex in the centroid of the NP under study, and that of an ideal hexagonal array pattern; essentially, *n* · 60^°^ is subtracted from the measured angle where *n* is the rounded-off integer of the measured angle divided by 60^°^. *x̅_Area_* and *x̅_Max FD_* are NP size indicators, *x̅_FF_* and *x̅_Ellips_* are shape features, whereas *s_d_* and *s_θ_* to some extent indicate how well the array pattern is maintained after the SMNPG. Δ*N*/*N*_0_, *S_d_*, and *S_θ_* are, however, dependent on both the size and shape of the NPs as well as the quality of the array pattern. Δ*N*/*N*_0_ will be further affected by the orientation of the SEM acquisition area in relation to the array lattice.

SE measurements [Woollam RC2-XI, photon energy (*E*) range = [0.7, 4.4] eV, angle of incidence 65^°^] were conducted in the center of the DoE samples with the probe spot along the diagonal running through points IP1–3. Au NP arrays on a substrate surface can optically be described in terms of an effective medium with an effective layer thickness (*t_eff_*) and relative permittivity (*ϵ* = *ϵ*_1_ + *iϵ*_2_). *t_eff_* and *ϵ* were estimated by fitting an oscillator model, consisting of a PSemi-M0, a PSemi-Tri, and ≤3 Gaussian oscillators, to the SE measurement data. In *ϵ*_2_, the background is represented by the PSemi-M0 and a wide Gaussian centered at high (>4 eV) photon energies (*E*) while the PSemi-Tri and remaining Gaussians correspond to the LSPR. The LSPRs of the NP arrays were characterized using automated peak detection [the findpeaks function] in MATLAB on the *ϵ*_2_ contribution of the sum of the LSPR oscillators. The extracted features were the LSPR peak position (*E_LSPR_*), amplitude (*ϵ*_2, *LSPR*_), and full width at half maximum (FWHM). Two seed particle array reference samples per parent wafer were equally analyzed for comparison.

### 2.7 Characterization of the Growth Media without Seed-Particle Arrays

During the unseeded DoE scheme, the growth media were recorded using a digital camera setup. Image frames were converted to grayscale and analyzed at a 2 s interval, starting 20 s after H_2_O_2_ addition to allow reagent mixing to complete. The mean pixel intensity (*I*) was then calculated in a fixed image window centered on the transparent PP beaker. To track changes over time and enable cross-sample comparisons, the relative intensity change Δ*I*(*t*)/*I*_0_ ≡ [(*I*(*t* = 0 s) – *I*(*t*))/*I*(*t* = 0 s)] was calculated. Functions of the form *f*(*t*) = *A* + *K*{1 + exp [ – (*t* – *t*_0_)/*τ*]}^−1^ were then fitted to the Δ*I*(*t*)/*I*_0_ data, with *A*, *K*, *τ*, and *t*_0_ as simultaneous fitting parameters.

### 2.8 Characterization of Samples Grown without Agitation

These samples were characterized equivalently to those in the sample DoE scheme with two exceptions. First, the DIA pipeline was modified so as to analyze and pool the results of two images acquired at 400 k magnification per inspection site. Second, multiple angles of incidence were used (i.e., {65, 70, and 75}^°^) during the SE data acquisition.

### 2.9 Statistical Modeling of Extracted Features

Multiple linear regression (MLR) with backward elimination^33^ (significance level, *α*= 0.05 to exclude) of independent variables was performed for several of the extracted features using the statistical software Minitab 17. This method entails an automated, iterative modeling procedure starting with the full model containing the reagent concentrations *c_citrate_*, *c*_*HAuCl*4_, and *c*_*H*2*O*2_ as well as their interaction and second order terms. Sequentially, the least significant variable, that is, the one with the highest *p*-value, is then eliminated one at a time until all remaining variables have a *p*-value smaller than *α*.^33^ No boundary conditions were imposed, and the independent variables were standardized (i.e., subtraction of the mean and division with the SD were performed) prior to modeling to reduce effects of multicollinearity.^33^ In instances of *R*^2^ < 80% (*R*^2^ defined below) and non-normally distributed residuals, an MLR was also conducted on the natural logarithm of that response variable. If substantial improvement was obtained, the model with the transformed response was adopted. When modeling the DIA-extracted features, the seed particle reference samples were only included in the dataset for *s_d_* and *s_θ_* (see definitions above) as the SEM images of the references were acquired at a lower *V_acc_* (1 kV). *R*^2^ (ratio of explained variance to total variance), adjusted *R*^2^ (*R*^2^ adjusted for the degrees of freedom in the model), and *R*_*pred*_^2^ (a measure of the predictive strength of the model) are the goodness of fit parameters used to quantify the fit and applicability of the models.^33^ The *R*^2^ contribution of each independent variable included in the model to the total *R*^2^ is also given to highlight its importance to the model.

## 3 Results and Discussion

### 3.1 DoE Scheme for SMNPG

The SEM structure verification of the loaded BCP templates reveals highly ordered patterns of hexagonally, nonclose-packed, AuCl_4_^–^-loaded P2VP domains in a PS matrix (Figure S1a–h). Subsequent ashing of these structures generates highly ordered Au seed particle arrays on the Si substrate (Figure S1i–q) with NPs of uniform size and shape (Table S1). The average values of the maximum Ferret diameter (*x̅_maxFD_*), ellipticity (*x̅_Ellips_*), and center-to-center interparticle distance (*x̅_d_*) are approximately 9, 1.1, and 41 nm, respectively. *x̅_Ellips_* is likely an overestimate though, due to the small NP size and finite pixel resolution. A small fraction of vacant lattice positions can be seen on the reference samples originating from one of the parent wafers (Figure S1j,n), possibly due to defects in the BCP template or insufficient precursor access during the loading procedure. The areal seed particle density on this wafer is 5.5% lower than the average of fully covered wafers, but out of the image-extracted features, this is only expected to affect the fractional areal coverage (*ϕ*) in any significant way.

**Figure 3:**
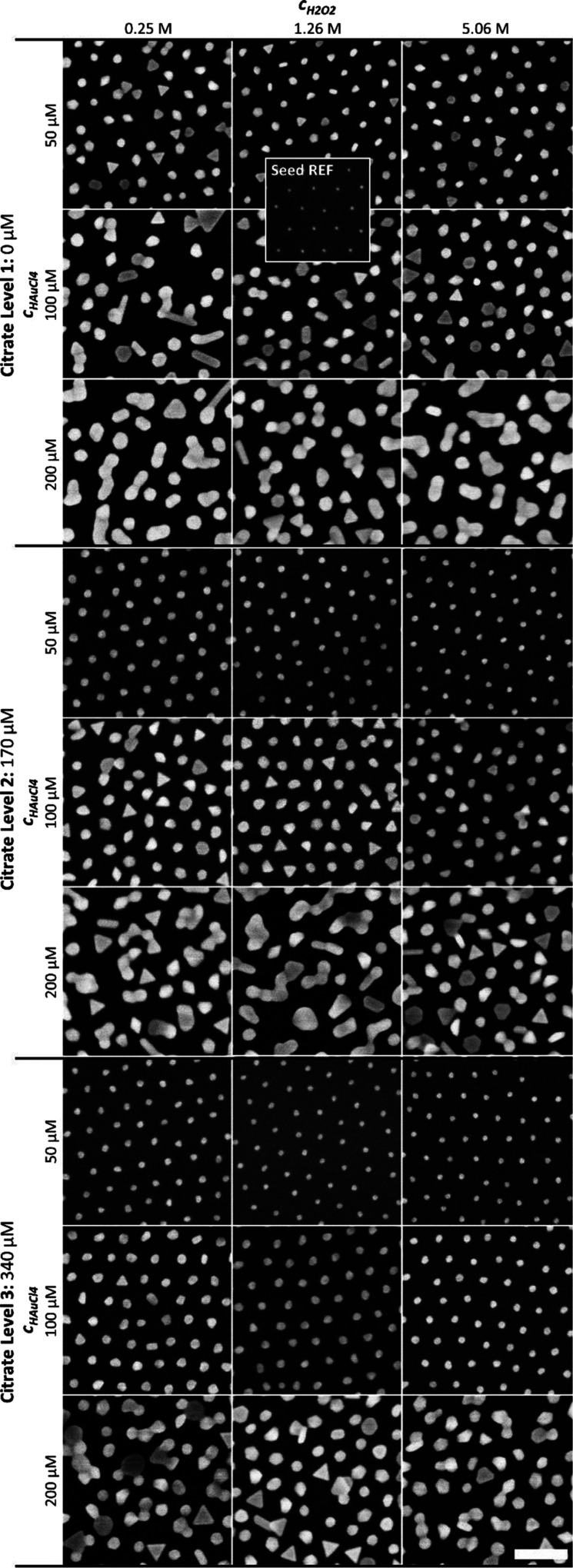
Compilation of SEM images acquired at the center (IP2) of the DoE samples after the SMNPG procedures. The scale bar equals 100 nm.

Seeded growth with agitation results in a macroscopically uneven deposition, as evident from visual inspection (Figure S2a), comparison of the SEM images (Figures S3–S5), and the affiliated DIA-extracted feature values (Table S2, Section I and Table S3). For example, taking only the DoE CP replicates into account, the average cross-sectional area (*x̅_area_*) of the NPs in the opposite corner points (IP1 and IP3) is 73% higher and 32% lower, respectively, than that in the middle point (IP2). An Au precursor mass-transport limitation of the deposition process in combination with growth medium agitation is the likely cause considering the vastly lower concentration of HAuCl_4_ compared to H_2_O_2_ throughout the DoE scheme. Such a mass-transport limitation would make the deposition highly sensitive to the local hydrodynamic conditions, which is consistent with the deposition pattern observed here. Drawing meaningful conclusions regarding the dependencies of the DIA- and SE-extracted features on the reagent concentrations is, nonetheless, still believed to be possible by exclusively analyzing the results obtained in the middle inspection point (IP2). The hydrodynamic conditions ought to vary the least between the samples here. The SE measurement spot was centered on this point. A compilation of SEM images from all the DoE samples shows that our DoE scheme covers the generation of NPs of various sizes, shapes, and degrees of interconnectedness. This suggests that the DoE reagent concentration ranges used were sufficiently broad here (Figure 3).

A straightforward way of probing the strength of linear relationships between the extracted features internally and the reagent concentrations is to compile a correlation matrix stating the pairwise Pearson correlation coefficients (*ρ*) (Figure S6). A strong correlation (|*ρ*|≥ 0.73) between *c*_*HAuCl*4_ and all DIA-extracted features, which in turn are all strongly correlated with each other (|*ρ*|≥ 0.78), can be observed. The positive correlations between *ϕ*, *x̅_Area_*, *x̅_Max FD_*, and *c*_*HAuCl*4_ are intuitive as the three features are all indicators of the amount of material deposited and *c*_*HAuCl*4_ dictates the availability of the Au precursor. The correlations between the DIA features and *c*_*H*2*O*2_ are very weak (|*ρ*|≤ 0.15), whereas those toward *c_citrate_* are stronger (0.17 ≤ |*ρ*| ≤ 0.51) in the probed parameter space. The former is likely related to the fact that *c*_*H*2*O*2_ ≫ *c*_*HAuCl*4_ even for the lowest *c*_*H*2*O*2_ DoE level used. The strong relationship between the SDs *s_Area_*, *s_Max FD_*, *s_FF_*, and *s_Ellips_* and their corresponding means implies a diversifying NP population, in terms of size and shape, with increasing growth.

To investigate the potential presence of more complex dependencies of the extracted sample features, we also performed modeling using MLR with backward elimination of independent variables on each DIA feature, as described in the Experimental Section.^33^ The significant independent variables, their effects, and relative importance are summarized in Table 1; the numerical values of the fitted coefficients are listed in Table S4. Decent statistical models, with adjusted explained variance ratio *adj*. *R*^2^ ≥ 76.4% and predictive strength *R*_*pred*_^2^ ≥ 74.3%, are obtained, and we observe that the *R*^2^ contribution of *c*_*HAuCl*4_ (and its square term) completely dominates in these models. *c_citrate_* is a significant but limited-influence variable in all but the model concerning the effective optical thickness. Meanwhile, *c*_*H*2*O*2_ only provides a marginal explanatory contribution in two of the models and is otherwise insignificant.

**Table 1:**
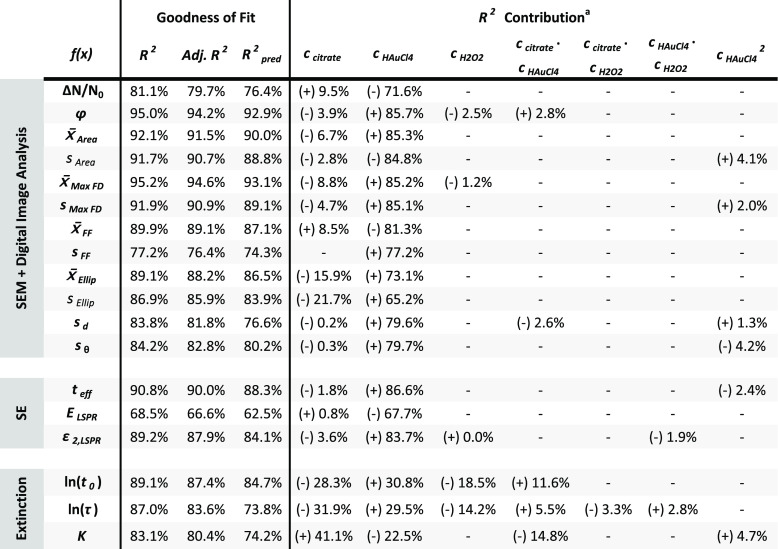
Extracted Features Modeled Using Multiple Linear Regression^a^

a *R*^2^-contribution of the significant linear (*c*_citrate_, *c*_HAuCl4_ and *c*_H2O2_), interaction (*c*_citrate_ · *c*_HAuCl4_, *c*_citrate_ · *c*_H2O2_ and *c*_HAuCl4_ · *c*_H2O2_ ) and square (*c*_HAuCl4_^2^) terms to the total *R*^2^-value of the models. The contributions from all significant terms add up to the total *R*^2^-value of the model. The sign of the coefficients are stated within the parenthesis. The reference samples are included in the modeling of *s*_d_, *s*_θ_ and the SE features.

**Figure 4:**
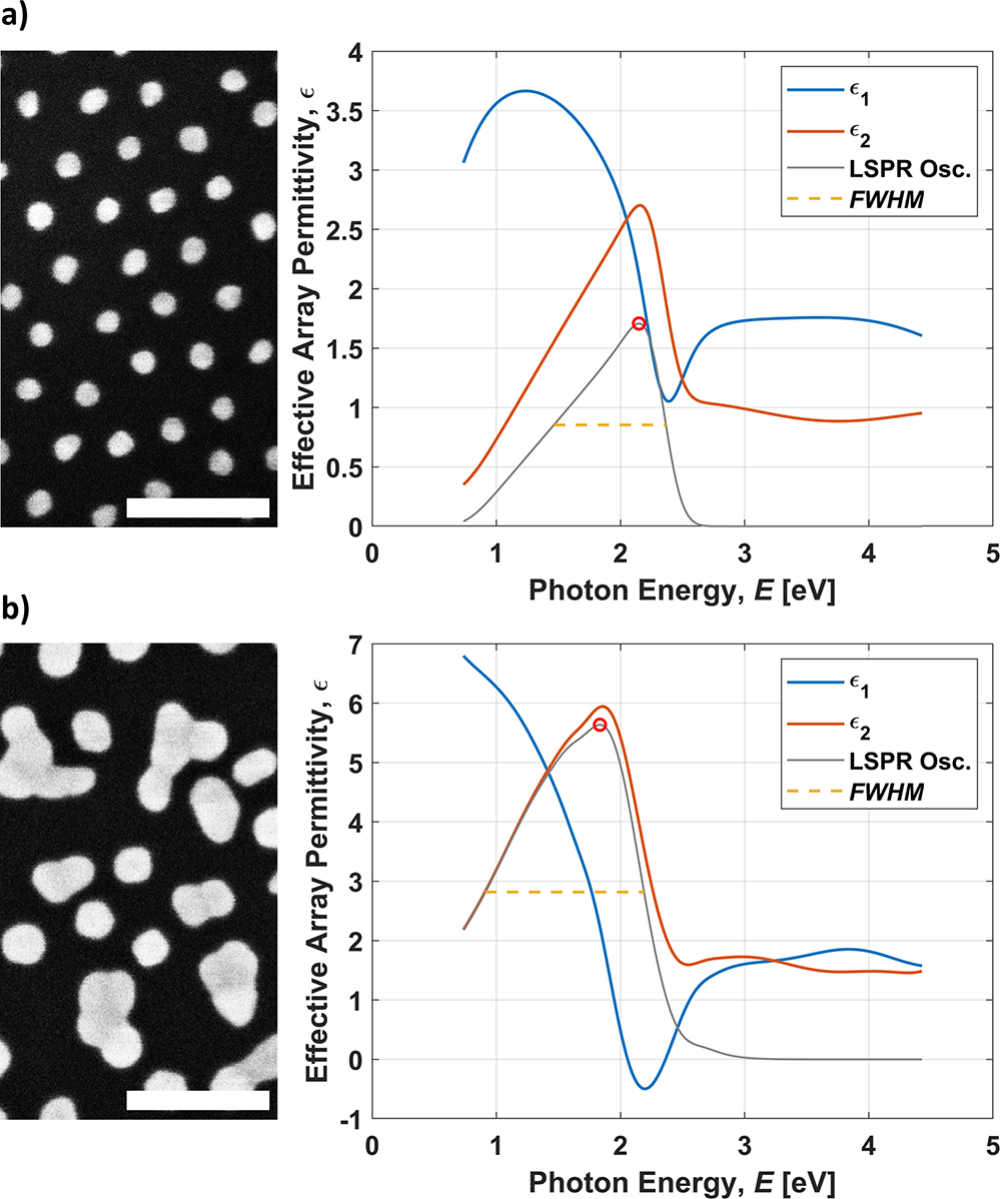
SEM images and plots of the effective array permittivity *ϵ* from two representative samples post-SMNPG with (a) moderately sized well-separated NPs and (b) large polydisperse NPs. The effective medium thickness (*t_eff_*), peak position (*E_LSPR_*), amplitude (*ϵ*_2, *LSPR*_), and width (*FWHM*) of the oscillator functions attributed to the LSPRs are {*t_eff_* = 59 Å, *E_LSPR_* = 2.15 eV, *ϵ*_2, *LSPR*_ = 1.71, and *FWHM* = 0.90 eV} and {*t_eff_* = 190 Å, *E_LSPR_* = 1.83 eV, *ϵ*_2, *LSPR*_ = 5.64, and *FWHM* = 1.28 eV} for panels (a) and (b), respectively. *E_LSPR_* and *ϵ*_2, *LSPR*_ are the coordinates indicated by the red circles. The scale bars equal 100 nm.

As many applications of Au NPs involve optics, photonics, or plasmonics, it is of great interest to also characterize the optical response of the resultant NP arrays using SE. The energy-resolved relative permittivity (*ϵ* = *ϵ*_1_ + *iϵ*_2_) of the effective media formed by the NP arrays (Figures S7–S11) is of particular interest as it can be used to characterize the collective LSPRs—as detailed in the Experimental Section (Table S2, Section II). The SE results are, however, likely affected by the deposition gradient across the SE measuring spot, which depending on incidence angle can extend for up to 10 mm. Nonetheless, both correlations and MLR models were calculated for the SE-extracted effective thickness (*t_eff_*), the plasmon peak position (*E_LSPR_*), amplitude (*ϵ*_2, *LSPR*_), and width (FWHM), with the exception of FWHM, which could not be obtained for all DoE samples due to limitations in the spectral range. The correlations between {*E_LSPR_*, *ϵ*_2, *LSPR*_, and *t_eff_*} and *c*_*HAuCl*4_ are moderate to strong (|*ρ*| ≥ 0.67), while the corresponding values for *c_citrate_* and *c*_*H*2*O*2_ are weak (|*ρ*| ≤ 0.34) and very weak (|*ρ* | ≤ 0.11), respectively (Table S6). At most, marginal effects of *c_citrate_* and *c*_*H*2*O*2_ on the SE features are also suggested by the MLR models (Table 1). *c*_*HAuCl*4_ on the other hand confers a strong explanatory power, which is readily understood from the fact that a higher *c*_*HAuCl*4_ enables larger particles and thereby prominently affects all of the SE features. According to our MLR models, a higher *c*_*HAuCl*4_ is affiliated with an increase of *ϵ*_2, *LSPR*_ and a decrease of *E_LSPR_* (i.e., a redshift of the LSPR peak), which is consistent with what would be expected, as larger NPs have a higher polarizability and support longer wavelength resonances.^34^ The nature of the LSPR peak can, however, be affected by more than just the size and shape distributions of the individual NPs. Some degree of modulation is also conceivable due to interparticle near-field coupling, the strength of which decays on a characteristic length scale of approximately 0.4 times the radius of the NPs.^29^ This phenomenon might be applicable to samples for which the initial array order is not maintained and where large, irregularly shaped NPs can be observed in close proximity, leading especially to an increased peak width in the measurements. The decay of the array order is, at least in part, likely attributable to the vigorous agitation of the growth media employed in the DoE scheme. An example of the SE-analyzed optical properties of two distinctively different NP arrays after seeded growth is shown in Figure 4.

### 3.2 DoE Scheme without Samples

Although the surface-supported NPs are of primary interest in this study, the tendency for HN in solution is important as it entails parasitic consumption of the available Au precursor and leads to reduced and less resource-efficient seeded growth. Hence, to investigate trends in the onset and severity of HN, the full DoE scheme was repeated using growth media without samples and an extended run duration of 15 min. A digital camera setup was used to optically monitor the solutions. In addition to compiling image collages of the media side-by-side at a set of specific time coordinates (Figures S12–S14), the relative change in mean grayscale pixel intensity [Δ*I*(*t*)/*I*_0_] was also calculated and tracked over time (*t*) for each run (Figure S15). Δ*I*(*t*)/*I*_0_ is here devised as a proxy for the combined extent of undesirable HN and growth of NPs in solution. This is motivated by the fact that suspended colloidal NPs will absorb and scatter light, causing the recorded pixel intensity to drop and Δ*I*(*t*)/*I*_0_ to increase commensurately. Next, Δ*I*(*t*)/*I*_0_ was modeled by fitting functions of the form *f*(*t*) = *A* + *K*{1 + exp [ – (*t* – *t*_0_)/*τ*]}^−1^ to the data from each run (Figures S16–S19 and Table S2, Section III). The sigmoid functional form captures the behavior of Δ*I*(*t*)/*I*_0_ very well except for in a few instances of discontinuity artifacts and/or a declining asymptotic plateau. Gas evolution in the medium, stemming from the NP-catalyzed decomposition of H_2_O_2_, is the likely cause of these artifacts as generated bubbles of varying numbers, sizes, and residence times can cling to the beaker wall (Figures S13 and S14). Although no gas bubble formation on the seed-decorated sample surface was observed in any of our sample DoE runs, if it would occur, it might interfere with precursor access. Thus, caution in using excessively high *c*_*H*2*O*2_ might be warranted. The fitting parameters *t*_0_ and *τ* are related to the onset and duration of the Δ*I*(*t*)/*I*_0_ change, respectively, whereas *A* + *K* is the value of the asymptotic plateau of *f*(*t*), that is, as *t* → ∞. However, as *A* merely accounts for very minor curve offsets, *K* is the parameter of interest to gauge the magnitude of change. All other things being equal, it is desirable for *t*_0_ and *τ* to be large while *K* ideally should be small as this would correspond to a late onset of a slow and low magnitude change. Considering the correlation matrix (Table S6) and performing individual MLRs on *K*, *t*_0_, and *τ* (Table 1) suggest that either increasing *c_citrate_* or decreasing *c*_*HAuCl*4_ will cause a larger Δ*I*(*t*)/*I*_0_ change, which further will start earlier and occur faster. A high *c_citrate_*:*c*_*HAuCl*4_ ratio, moreover, results in blueish growth media, whereas more reddish solutions are obtained for lower ratios (Figures S12–S14). This is interpreted as a high *c_citrate_*:*c*_*HAuCl*4_ ratio being affiliated with more rapid and extensive HN, resulting in a higher number of small but growing colloidal NPs contributing to the optical extinction of the medium. This is the opposite of what we would expect merely on the basis of thermodynamics as the tendency for HN should increase with increasing redox potential (*E_r_*) of the system. The dependencies of *E_r_* on the reagent concentrations are given by the Nernst equation, , where *E_r_^o^*, *R*, *T*, *z*, *F*, and *Q_r_* are the standard redox potential, universal gas constant, temperature, number of electrons transferred in the reaction, Faraday constant, and reaction quotient, respectively. As *c*_*HAuCl*4_ occurs in the denominator of *Q_r_*, an increase ought to lead to a higher *E_r_*. The addition of citrate, on the other hand, is expected to mainly affect Au(III) complex speciation by acting as an alternative ligand to Cl^–^, thereby lowering *E_r_*. As both trends are at odds with our observations, we thus conclude that the rate of nucleation is limited by reaction kinetics rather than thermodynamics. Essentially, a higher conversion of AuCl_4_^–^ to Au(III)–citrate complex in the solution is believed to aid the electron transfer required for Au nucleation and subsequent growth, and the Au(III) complex speciation is determined by the *c_citrate_* : *c*_*HAuCl*4_ ratio. The reason that the observed effect of this ratio is so pronounced in our experiments is likely that the chosen *c_citrate_* and *c*_*HAuCl*4_ DoE levels are comparable in values. Obtaining rounder but smaller surface-supported NPs when using high *c_citrate_* : *c*_*HAuCl*4_ ratios is also consistent with having higher rates of nucleation and growth as adatom selectivity between different crystallographic planes and parasitic consumption of Au(III) is expected to decrease and increase, respectively. Citrate also has secondary effects, which might affect the final optical appearance of the growth media. First, negatively charged citrate ions adsorbed on the surface of NPs contribute to the electrostatic repulsion between NPs, reduce the rate of NP aggregation, and thus delay the redshift of the extinction peak. Second, it is conceivable that the adsorbed citrate could change the immediate dielectric environment of the NPs so as to influence optical extinction by the LSPR of NPs in solution. As for the effects of *c*_*H*2*O*2_, our MLRs suggest that an increase does not affect the magnitude of the Δ*I*(*t*)/*I*_0_ change but expediates its onset and potentially also hasten its pace. The limited effects of *c*_*H*2*O*2_ is readily explained by the fact that it is present in large excess throughout the DoE schemes.

We thus surmise that the rates of both nucleation and growth are kinetically limited. The latter is evident from the uneven deposition seen in our sample DoE scheme, which strongly suggests that mass transport of the Au(III) complex is the limiting factor.

### 3.3 Identification of a Suitable Seeded Growth Process Window

**Figure 5:**
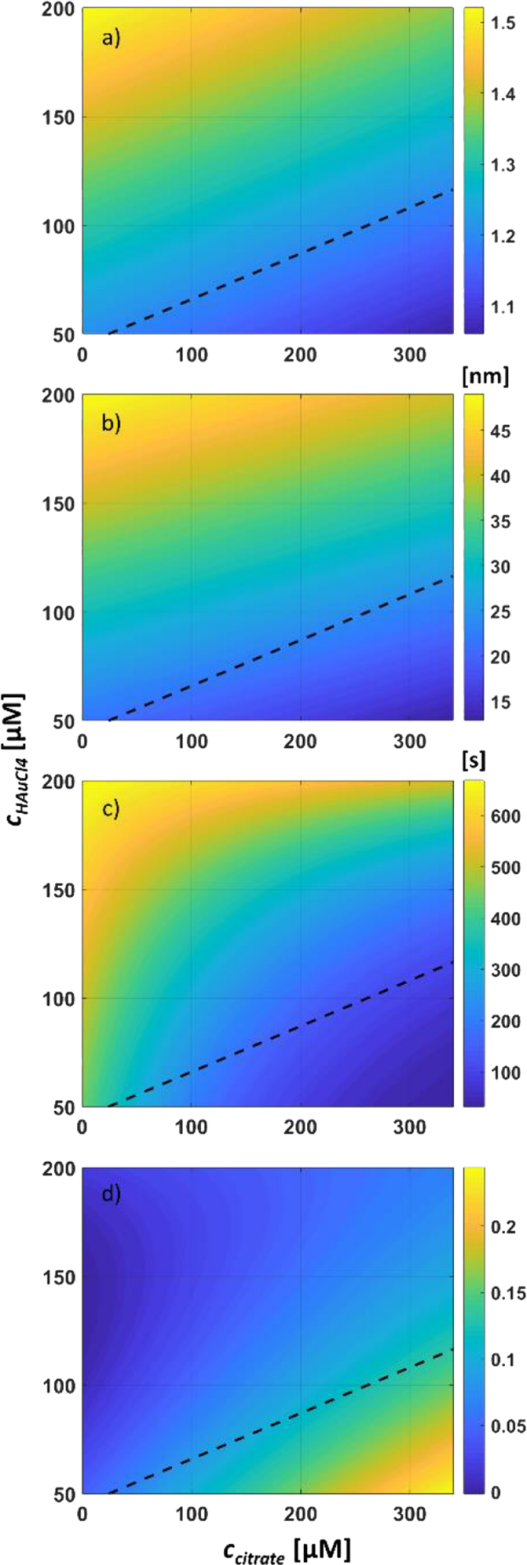
Heatmaps of modeled (a) NP mean ellipticity (*x̅_Ellips_*), (b) NP mean maximum Ferret diameter (*x̅_Max FD_*), unseeded medium Δ*I*(*t*)/*I*_0_ change, (c) onset (*t*_0_), and (d) magnitude (*K*). The dashed lines represent *x̅_Ellips_*= 1.2, thereby defining a process window for obtaining quasi-spherical NPs in the bottom right corner.

Heatmaps of our MLR-modeled *x̅_Ellips_*, *x̅_Max FD_*, *t*_0_, and *K* are shown in Figure 5 with the aim to visualize the growth process window in which quasi-spherical surface-supported NPs can be obtained and further indicate what we can expect in terms of particle size and extent of HN. Here, we have somewhat arbitrarily defined an array of quasi-spherical NPs as one having a *x̅_Ellips_* ≤ 1.2. This leads to the process window partially shown in Figure 5a, defined by {*c*_*H*2*O*2_ ∈ [0.25, 5.06] M, *c*_*HAuCl*4_ ∈ [50, 117] μM and *c_citrate_* ≥ 4.75 · *c*_*HAuCl*4_ – 214 μM}. The middle *c*_*H*2*O*2_ DoE level was used in plotting *t*_0_ here, but equivalent plots of *t*_0_ and *τ* for all three DoE levels are shown in Figure S20. The result shown in Figure 5a shows that an increase of *c_citrate_* (for a given *c*_*HAuCl*4_) will result in more circular top-view cross sections, but from Figure 5b, it is observed that it will also lead to smaller NPs; the latter is due to increased HN in the growth medium with its affiliated parasitic consumption of HAuCl_4_. In Figure 5c, the middle *c*_*H*2*O*2_ DoE level (1.26 M) was used for plotting the HN onset time *t*_0_ with consideration of gas bubble formation and reagent consumption. For completeness, citrate, in the absence of H_2_O_2_, does not seem to be able to reduce Au(III) to Au(0) and cause HN at an appreciable rate under these conditions; this was demonstrated by preparing an unseeded growth medium with a high *c_citrate_*:*c*_*HAuCl*4_ ratio but without any H_2_O_2_ and letting it run for 1 h. As no change in Δ*I*(*t*)/*I*_0_ was observed, this suggests that H_2_O_2_ is the dominant reducing agent in this reaction system (Figure S21).

For verifying reproducibility, a cross-sample comparison of the four DoE CP replicates reveals virtually identical NPs, in terms of shape and size, although the array order has been maintained to a varying degree (Figure S22a–e,g). As for HN of the corresponding unseeded media, the curves fitted to Δ*I*(*t*)/*I*_0_ have similar rise times (*τ*) ([45, 60] s) and magnitudes (*K*) ([0.052, 0.080]) but more varied onsets (*t*_0_) ([196, 381] s) as would be expected of a process containing stochastic elements (Figure S22f).

### 3.4 Seeded Growth without Agitation

**Figure 6:**
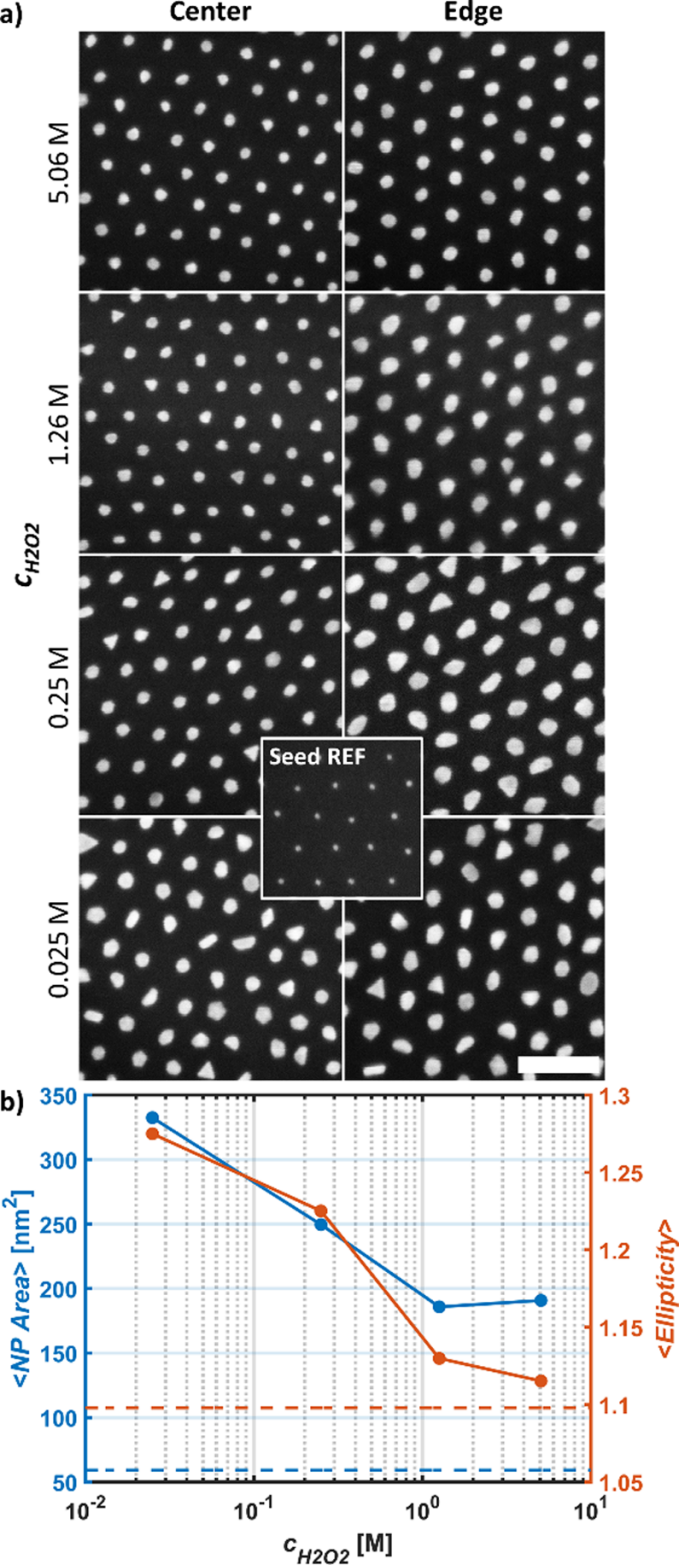
(a) Evaluation of progressively decreasing *c*_*H*2*O*2_ as shown in the figure in the absence of growth medium agitation. *c*_*HAuCl*4_ = 100 μM and *c_citrate_* = 340 μM were used for all samples. The SEM images were acquired close to the sample center and 100 μm from the sample edge, respectively. Scale bar equals 100 nm. (b) Plots of the mean NP area and mean ellipticity of the surface-supported NPs (in sample center) on the samples in panel (a). Dashed lines represent the corresponding values for the initial seed arrays.

One of the main takeaways from our DoE scheme is that consistent hydrodynamic conditions over the seed-decorated surface are required if a uniform degree of deposition is to be obtained in the probed reagent parameter space (Figure S2). The most straightforward way of approximating this is to forgo any growth medium agitation. As it is also of interest to determine to which extent *c*_*H*2*O*2_ can be dialed back, to minimize gas bubble formation and reagent consumption, we chose to run a sample series without agitation using the highest *c_citrate_* (340 μM) and middle *c*_*HAuCl*4_ (100 μM) DoE levels while progressively decreasing *c*_*H*2*O*2_ {5.06 M, 1.26 M, 0.25 M, 0.025 M}. To gauge the deposition uniformity, SEM images were acquired at the sample center as well as 100 μm from the edge closest to the beaker bottom during SMNPG. The NPs at the edges were consistently somewhat larger than those in the sample center, which is partly to be expected due to higher levels of medium turbulence here during sample handling (Figure 6a).

DIA suggests that the size and ellipticity of the NPs increase with decreasing *c*_*H*2*O*2_ (Figure 6b, Table S5), however, with the caveat that these samples differed slightly in size. The size difference was estimated by weighing the samples, assuming a uniform wafer thickness, and was found to be marginal (<4%) (Table S5). Although using the highest *c*_*H*2*O*2_ yielded the most spherical NPs, one instance of gas bubble nucleation and growth was observed on the sample surface under these conditions, which could potentially be detrimental locally. All samples appear to be uniform after SMNPG under visual inspection (Figure S23 a–d), and SE optical characterization and feature extraction reveal well-defined LSPR peaks (Figure S24). We thus conclude that no agitation is preferable (in lieu of more sophisticated means of ensuring uniform, well-defined hydrodynamic conditions) when using this SMNPG protocol. The result shown in Figure 6b further suggests that *c*_*H*2*O*2_ might have a larger impact on the size and shape of the resultant NPs than our DoE scheme-based MLR models imply as *c*_*H*2*O*2_ was not found to be a significant variable in either *x̅_area_* or *x̅_ellip_*.

In addition to the experiments described above, we also investigated the effects of using PVA instead of, or in conjunction with, citrate in the growth medium. We found that PVA can delay the onset of the Δ*I*(*t*)/*I*_0_ change in the absence of seed particles and that larger surface-supported NPs can be obtained after seeded growth when using a combination of citrate and PVA. Addition of PVA appears capable of increasing the Au precursor utilization by suppressing HN in solution but at the cost of slightly reduced roundness (higher *x̅_Ellips_*) of the surface-supported NPs. The experiments are described in detail in the Supporting Information (Supporting Information Part II).

## 4 Conclusions

A seeded growth protocol consisting of a rapid, one-step procedure conducted at RT is employed on arrays of surface-supported Au seed particles. With the goal to establish conditions for the growth of quasi-spherical NPs, a fully orthogonal DoE scheme is applied to identify a process window for concentrations of the three growth medium reagents, namely, HAuCl_4_, citrate, and H_2_O_2_ (i.e., *c*_*HAuCl*4_, *c_citrate_*, and *c*_*H*2*O*2_). Using SEM and DIA, various features related to the average size, shape, and array pattern quality are quantified for the resultant NP arrays. The extracted features are modeled as functions of the reagent concentrations using MLR with stepwise backward elimination of independent variables. By defining arrays of grown NP with a mean ellipticity ≤1.2 as quasi-spherical, a suitable reagent concentration window is identified and defined by {*c*_*H*2*O*2_ ∈ [0.25, 5.06] M, *c*_*HAuCl*4_ ∈ [50, 117] μM and *c_citrate_* ≥ 4.75 *c*_*HAuCl*4_ – 214 μM}. SE is further performed to study the optical properties of the resulting Au NP arrays. Features related to the LSPR are identified and extracted, and their dependencies on reagent concentrations are also modeled using MLR.

In addition to reagent concentrations, we find that uniform hydrodynamic conditions over the NP-decorated substrate surface are key to obtain a macroscopically even deposition. This is presumably due to a mass-transport limited supply of the Au precursor in the surface reaction. Completely forgoing agitation of the growth medium is a convenient way of approximating the required conditions.

As varying degrees of parasitic HN, seemingly dependent on the growth medium composition, were observed during the sample DoE scheme, the entire scheme was repeated using equivalent media without seeded substrates to map these tendencies. A proxy of the optical extinction in the media is tracked over time and then modeled in two steps so as to discern its dependencies on the growth medium composition. Increasing *c_citrate_*, for a given *c*_*HAuCl*4_, is thereby found to exacerbate HN likely due to the ability of citrate to aid the electron transfer kinetics by acting as a complexing agent and thus affecting Au(III) speciation. This is unfortunate as a high *c_citrate_* also has a quasi-spherical shape-preserving effect on the surface-supported NPs. One potential solution to this issue is the addition of PVA to the growth medium as we observed that PVA can effectively delay the observable onset of our measured proxy for HN. Suppressed HN and thereby higher HAuCl_4_ utilization might thus be possible with PVA but appears to come at the cost of slightly more irregularly shaped surface-supported NPs.

We have identified seeded growth conditions that achieve a uniform deposition over large surfaces while maintaining the quasi-spherical shape and the initial array order of surface-supported nanoparticles. Applications in catalysis, photonics, photovoltaics, plasmonics, and several other areas are conceivable.

## Supporting Information Available

SEM images of the Au precursor-loaded BCP templates and seed particle arrays, tabulated feature values extracted via DIA and SE, compilations of photographs and SEM images of samples post-SMNPG, correlation matrix of dependent and independent variables, MLR models of extracted features, plots of the SE-modeled *ϵ*(*E*), plots of *t*_0_ and *τ* for different H_2_O_2_ concentrations, unseeded DoE media side-by-side at specific timestamps, curve fits to Δ*I*(*t*)/*I*_0_ data from the DoE unseeded media, growth medium without H_2_O_2_, comparison of DoE CP replicates, SMNPG experiments using PVA instead of, or in conjunction with, citrate (PDF)

## Abbreviations

ALD: atomic layer deposition
BCP: block copolymer
CP: centerpoint
DI: deionized
DIA: digital image analysis
DoE: design of experiment
HN: homogeneous nucleation
*h*PS: *homo*polystyrene
IP: inspection point
LSPR: localized surface plasmon resonance
NP: nanoparticle
RT: room temperature (21 °C)
sccm: standard cubic centimeters per minute
SEM: scanning electron microscopy
SMNPG: seeded metal nanoparticle growth
SVA: solvent vapor annealing
PS-*b*-P2VP: poly(styrene-*block*-2-vinylpyridine)
PVA: poly(vinyl alcohol)
