# Supplementary material for: Process Window for Seeded Growth of Arrays of Quasi-Spherical Substrate-Supported Au Nanoparticles
Source: Langmuir. 2021 May 3;37(19):6032–41. doi: 10.1021/acs.langmuir.1c00693 (PMC8280595; doi:10.1021/acs.langmuir.1c00693)
Supplement: Supplementary file 1 — la1c00693_si_001.pdf [file la1c00693_si_001.pdf]

Process Window for Seeded Growth of Arrays of Quasi-spherical Substrate-Supported Au Nanoparticles

Björn Landeke-Wilsmark,[†] Leif Nyholm[‡] and Carl Hägglund*[,†]

[†]Division of Solar Cell Technology, Department of Materials Science and Engineering, Uppsala University, P.O. Box 35, 751 03 Uppsala, Sweden
[‡]Division of Inorganic Chemistry, Department of Chemistry – Ångström Laboratory, Uppsala University, P.O. Box 523, 751 20 Uppsala, Sweden

* carl.hagglund@angstrom.uu.se

Supporting Information (SI)

Contents

PART I: DoE and Alternative Means of Agitation Experiments
SEM structure verification of Au precursor loaded BCP template and seed arrays ....Figure S1
Summary of extracted data from the seed particle reference samples..................................Table S1
Compilation of photographs of the DoE samples post-SMNPG ....Figure S2
SEM image compilations of DoE samples acquired at IP1-IP3 ....Figure S3-5
Summary of data extracted from the DoE samples, at IP2, and the corresponding unseeded growth media ....Table S2
Summary of data extracted at IP1 and IP3 for the DoE samples ....Table S3
Correlation matrix of extracted features and reagent concentrations ....Figure S6
Summary of features modelled using multiple linear regression ....Table S4
Modelled t_{eff} and $\epsilon(\lambda)$ of DoE samples before and after SMNPG ....Figure S7-S11
Image collages of DoE unseeded media side-by-side at specific process timestamps..............Figure S12-S14
Compilation of $\Delta I(t)/I_0$ curves from DoE unseeded media..Figure S15
Curve fits to $\Delta I(t)/I_0$ data from DoE unseeded media ....Figure S16-19
Heatmaps of t_0 and τ for the c_{H2O2} DoE-levels ....Figure S20
Time evolution of unseeded growth medium without H_2O_2...Figure S21
Compilation of data and images from the DoE centerpoint replicates....................................Figure S22
Summary of DIA-extracted Data After SMNPG with No Growth Medium Agitation ....Table S5
Compilation of photographs of samples used in the follow-up experiments ....Figure S23
Modelled t_{eff} and $\epsilon(\lambda)$ of the alt. agitation samples after SMNPG ....Figure S24

PART II: Use of PVA Instead of, or in Conjunction with, Citrate During SMNPG.....p. 28
$\Delta I(t)/I_0$ curves from unseeded media containing citrate and/or PVA ....Figure S25
Compilation of SEM images of samples for which PVA was used during SMNPG....................Figure S26
Tabulated DIA-extracted data from samples for which PVA was used during SMNPG.............Table S6
Plotted $\bar{x}_{area}$ and $\bar{x}_{Ellips}$ from samples for which PVA was used during SMNPG ....Figure S28

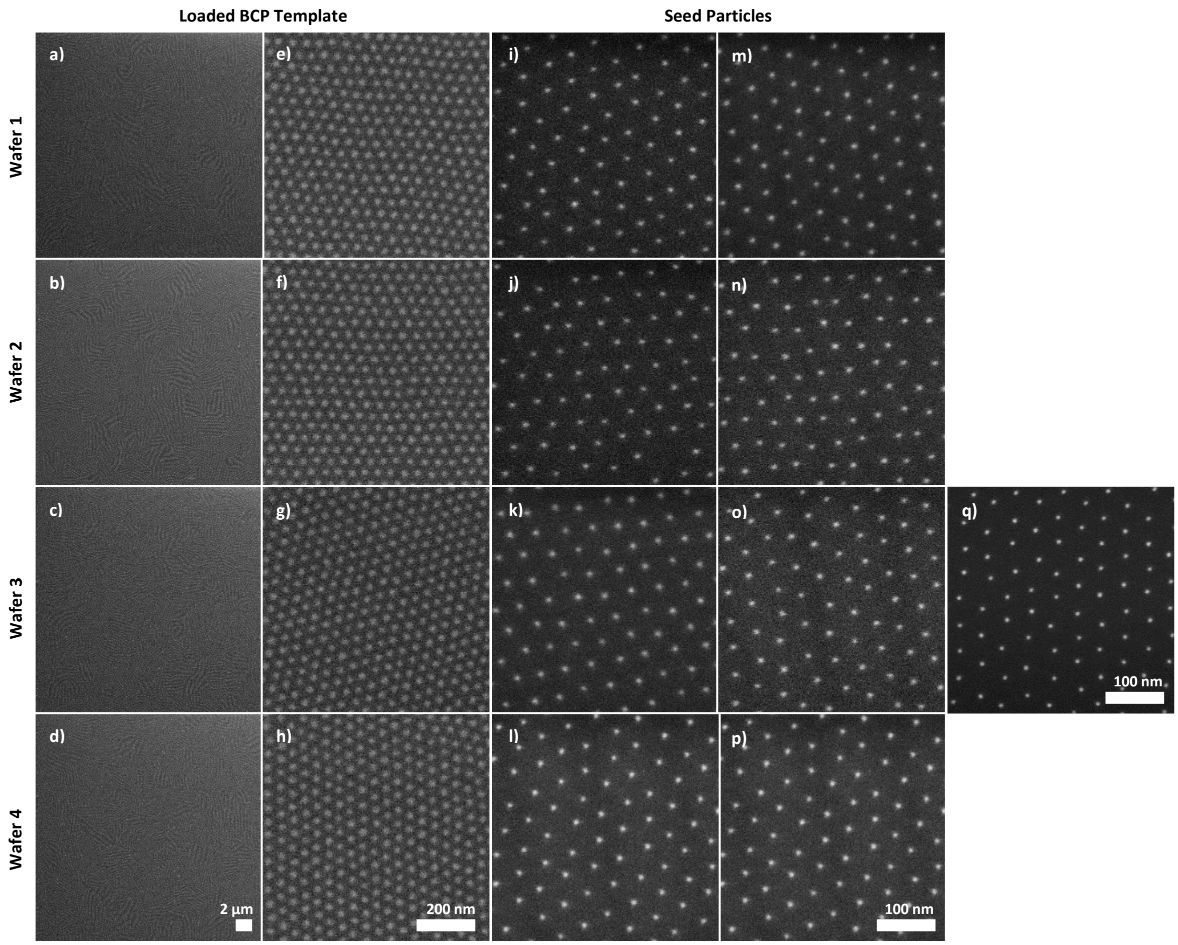

Figure S1: SEM-inspection at 5k (a-d) and 200k (e-f) magnification of sample parent wafers after the $HAuCl_4$-loading of the BCP template. Panels i-p constitute a verification of the seed particle arrays after ashing, dicing and stripping the S1813 protective coating. Two seed reference samples per parent wafer were inspected. $V_{acc} = 1\ kV$ was used in panels a-p. The image in panel q is from a spare edge piece from wafer 3 inspected at $V_{acc} = 5\ kV$ – note the difference in apparent particle size and noise level.

Table S1: Summary of Extracted Data from the Seed Particle Reference Samples

	**SECTION I** SEM + Digital Image Analysis													**SECTION II[a]** Spectroscopic Ellipsometry				
**Wafer**	***N***	**φ [%]**	**$\bar{X}_{Area}$ [nm^2]**	s_{Area} [nm^2]	**$\bar{X}_{Max\ FD}$ [nm]**	**$s_{Max\ FD}$ [nm]**	**$\bar{X}_{FF}$**	**s_{FF}**	**$\bar{X}_{Ellip}$**	s_{Ellip}	**$\bar{X}_d$ [nm]**	**s_d [nm]**	**s_θ [°]**	**t_{eff} [Å]**	**E_{LSPR} [eV]**	**$\varepsilon_{2,LSPR}$ [ε_0]**	***FWHM* [eV]**	***MSE***
1	688	5.3	119	30	13	2.0	0.90	0.07	1.18	0.11	40.7	4.0	9.6	3.11	2.16	0.18	0.39	0.588
1	682	5.1	121	27	13	1.7	0.90	0.07	1.21	0.09	41.1	3.0	10.1	2.51	2.16	0.15	0.40	0.600
2	654	6.5	146	36	14	2.0	0.90	0.07	1.13	0.09	40.8	3.8	10.8	1.17	2.214	0.09	0.41	0.616
2	642	5.0	124	32	13	2.0	0.89	0.07	1.20	0.10	41.3	3.5	10.6	1.72	2.179	0.25	0.36	0.597
3	684	5.2	116	30	12	1.8	0.92	0.07	1.17	0.09	40.9	3.4	9.2	1.82	2.157	0.30	0.37	0.581
3	679	6.0	131	25	13	1.5	0.90	0.07	1.21	0.09	42.1	2.5	8.2	3.99	2.267	0.05	0.60	0.606
4	688	5.4	122	23	13	1.5	0.91	0.07	1.11	0.08	41.8	2.7	8.7	1.66	2.195	0.10	0.38	0.637
4	694	5.8	129	29	14	1.8	0.89	0.07	1.23	0.11	41.3	2.8	8.7	1.47	2.183	0.16	0.39	0.626
3	352[b]	2.5	59	14	8.8	1.1	0.93	0.04	1.10	0.07	40.7	2.9	10.9	-	-	-	-	-

Comment: ***N*** and ***φ*** are the number of counted NPs and the fractional area coverage of NPs respectively. $\bar{X}_{Area}$, $\bar{X}_{Max\ FD}$, $\bar{X}_{FF}$, $\bar{X}_{Ellips.}$ and $\bar{X}_d$ are the mean NP cross-sectional area, max Ferret diameter, fill factor and center-to-center interparticle distance respectively while s_{Area}, $s_{FD\ max}$, s_{FF}, $s_{Ellips.}$ and s_d are the corresponding standard deviations. s_θ is the standard deviation of the discrepancy between the angle spanned by the centroids of the two nearest neighbours and that of a perfect hexagonal geometry. t_{eff}, E_{LSPR}, $\varepsilon_{2,LSPR}$ and ***FWHM*** are the modelled effective medium thickness, LSPR peak position, magnitude and width respectively. ***MSE*** is the mean square error of the SE model. [a]Peak position, height and width were extracted from one sample from each parent wafer. [b]The DIA was perfomed on SEM-images aquired at V_{acc} = 1 kV and 200k magnification for all samples except the last entry when the images analyzed were taken at 5 kV and 400k magnification.

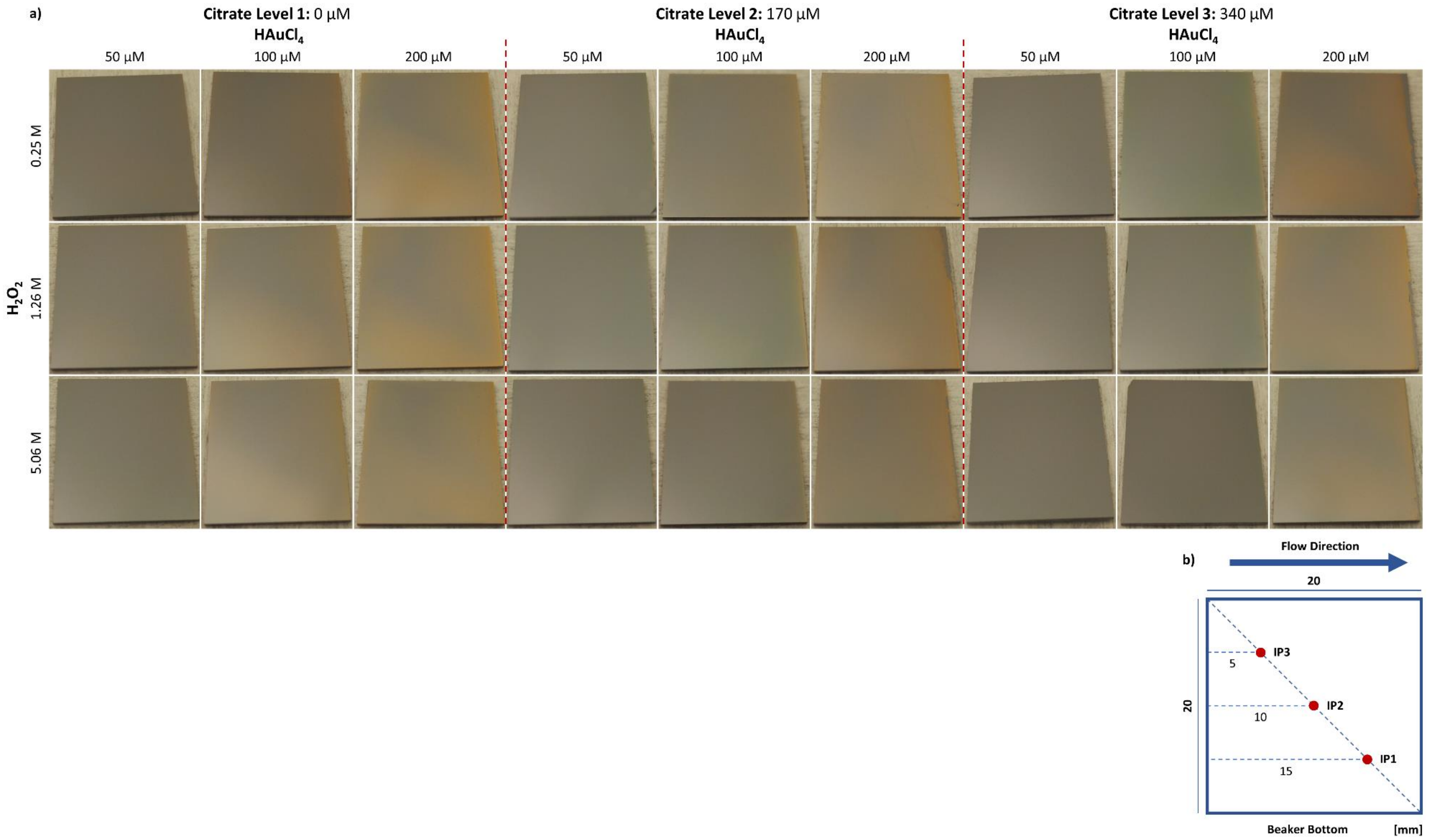

Figure S2: a) Compilation of photographs taken of the samples included in the DoE scheme after the SMNPG procedure. All samples are oriented in accordance with the legend in b). b) Positions of the inspection points on the sample surface and in relation to the flow direction of the growth medium during the DoE SMNPGs.

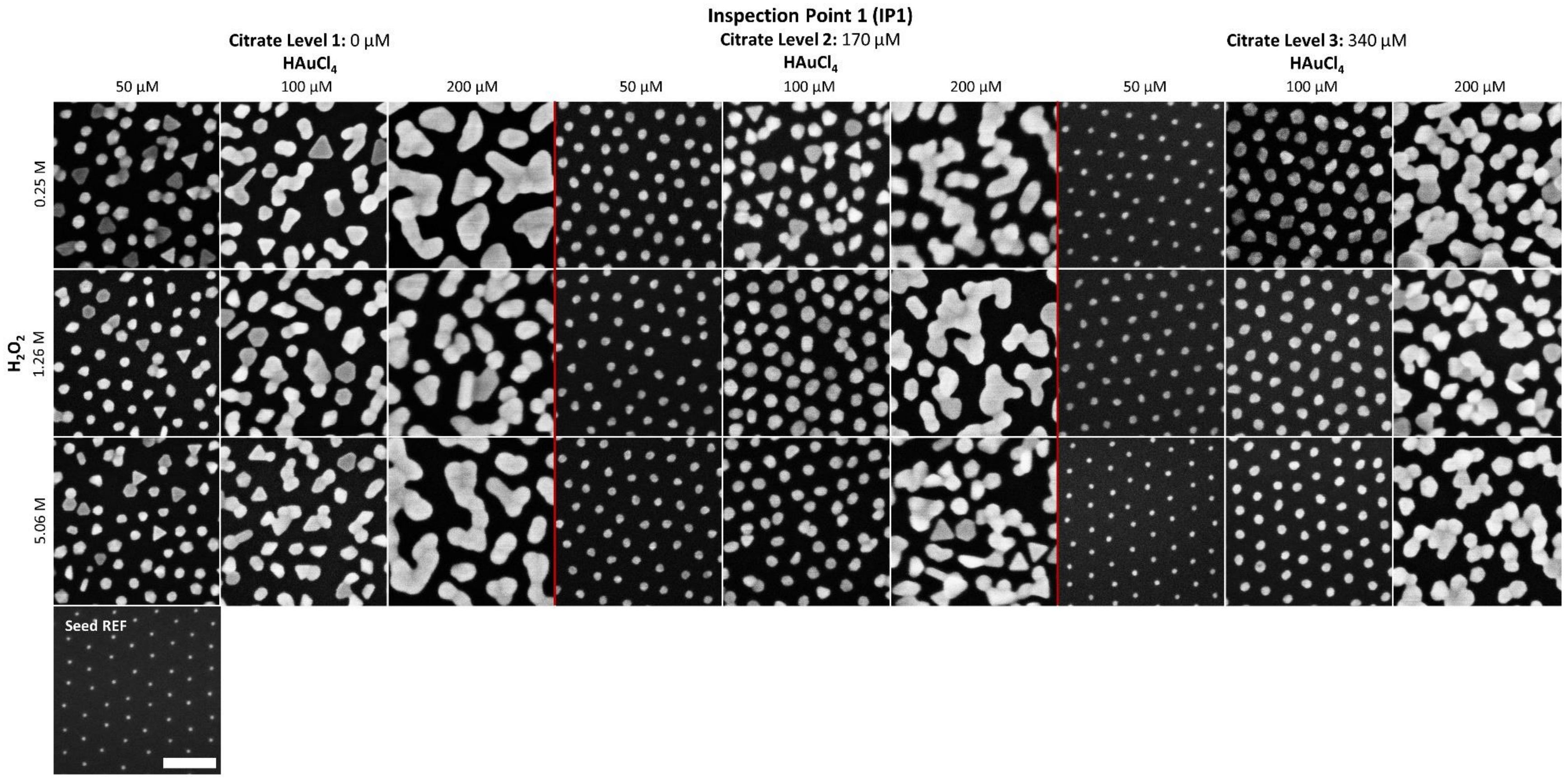

Figure S3: Compilation of SEM images, acquired at IP1, of the samples included in the DoE scheme. The seed reference sample was also inspected at $V_{acc} = 5\ kV$ and originates from parent wafer 3. The scalebar equals 100 nm.

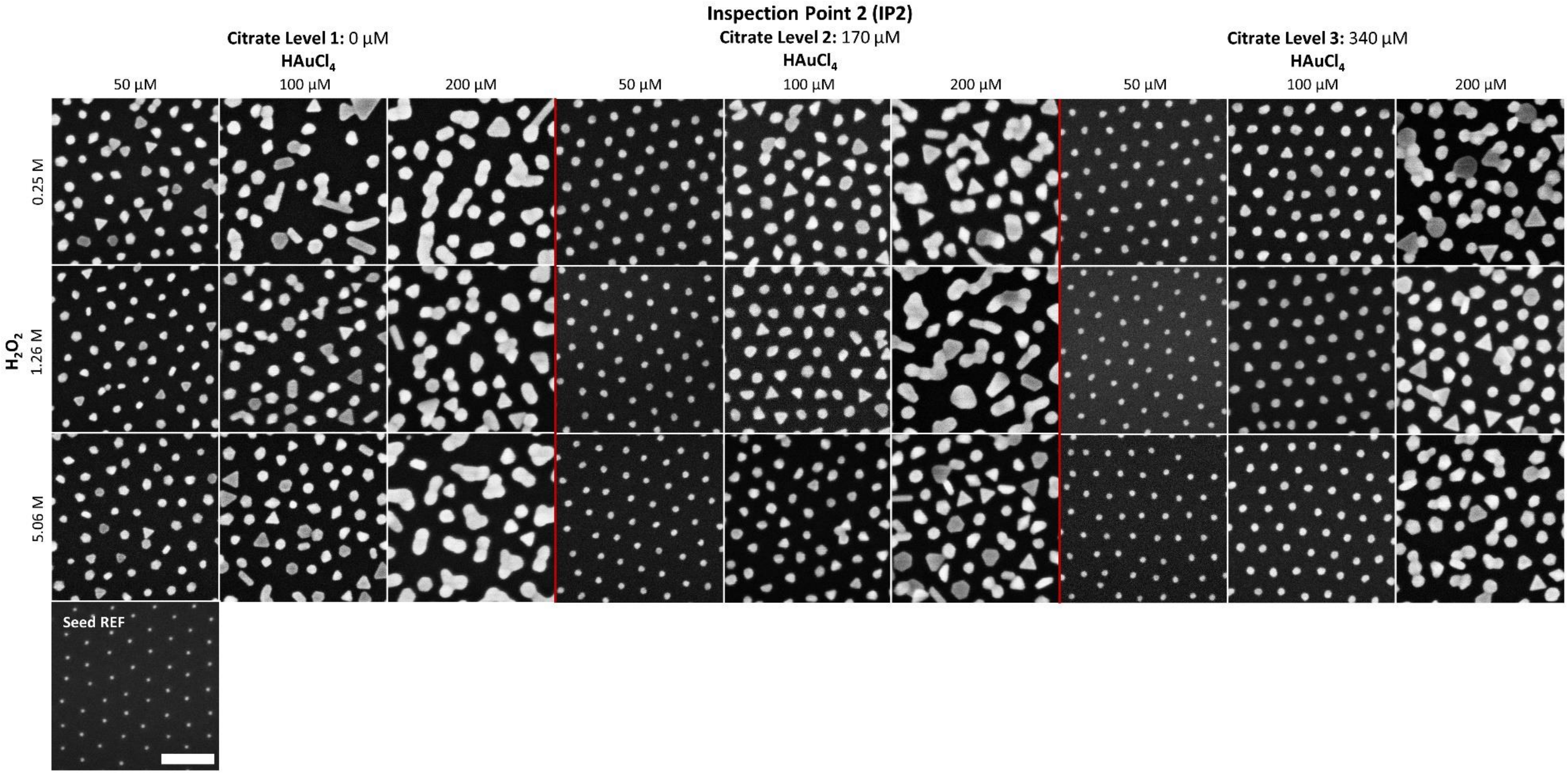

Figure S4: Compilation of SEM images, acquired at IP2, of the samples included in the DoE scheme. The seed reference sample was also inspected at $V_{acc} = 5\ kV$ and originates from parent wafer 3. This figure is identical to Figure 3 in the paper and is included here for completeness and ease of comparison. The scalebar equals 100 nm.

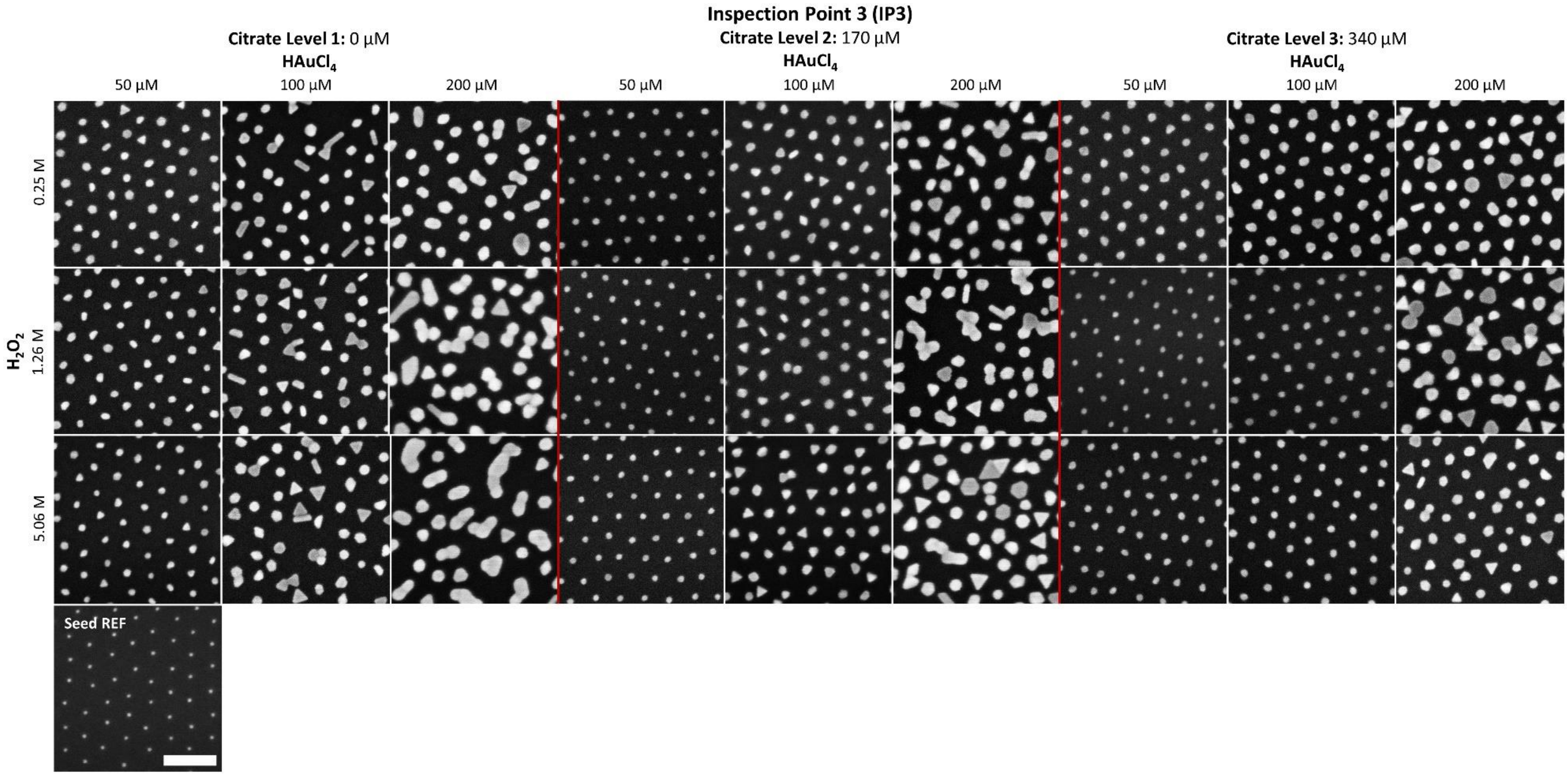

Figure S5: Compilation of SEM images, acquired at IP3, of the samples included in the DoE scheme. The seed reference sample was also inspected at $V_{acc} = 5\ kV$ and originates from parent wafer 3. The scalebar equals 100 nm.

Table S2: Summary of Data Extracted from the DoE Samples, at IP2, and the Corresponding Unseeded Growth Media

Inspection Point 2 (IP2) + Unseeded Media

Design of Experiment					SECTION I SEM + Digital Image Analysis												SECTION II Spectroscopic Ellipsometry					SECTION III Unseeded Media				
Wafer	Run	$c_{Citrate}$ [μM]	c_{HAuCl4} [μM]	c_{H2O2} [M]	$\Delta N/N_0$ [%]	φ [%]	$\bar{X}_{Area}$ [nm^2]	s_{Area} [nm^2]	$\bar{X}_{Max FD}$ [nm]	$s_{Max FD}$ [nm]	$\bar{X}_{FF}$	s_{FF}	$\bar{X}_{Ellip}$	s_{Ellip}	s_d [nm]	s_θ [°]	t_{eff} [Å]	E_{LSPR} [eV]	$\varepsilon_{2,LSPR}$ [ε_0]	*FWHM* [eV]	*MSE*	t_0 [s]	τ [s]	A	K	Adj. R^2
3	14	0	50	0.25	-3.6	15.7	361	110	23.0	4.8	0.89	0.07	1.21	0.24	4.7	13.9	81	1.98	2.38	1.11	0.691	549	105	0.00	0.032	0.97
4	17	0	50	1.26	-3.0	10.8	258	43	19.0	1.9	0.91	0.05	1.17	0.16	4.0	11.5	78	1.86	2.41	1.31	0.816	345	77	0.00	0.047	0.96
4	7	0	50	5.06	-3.9	12.5	290	58	20.2	2.7	0.90	0.06	1.16	0.20	4.7	13.5	76	1.99	2.38	0.94	0.647	241	36	0.00	0.057	0.99
3	28	0	100	0.25	-32.8	24.4	744	333	35.9	12.5	0.83	0.12	1.44	0.58	6.0	16.6	110	1.74	3.85	1.36	0.765	582	188	-0.01	0.029	0.94
4	18	0	100	1.26	-16.4	20.4	521	214	29.2	8.7	0.85	0.10	1.34	0.41	5.7	15.4	106	1.73	3.25	1.23	0.781	348	53	0.00	0.028	0.97
4	20	0	100	5.06	-14.6	20.1	508	231	28.8	9.3	0.85	0.11	1.35	0.45	5.2	15.2	102	1.81	2.98	1.31	0.866	218	82	0.00	0.025	0.91
3	21	0	200	0.25	-52.2	29.1	1228	727	47.2	19.8	0.81	0.13	1.51	0.56	9.3	16.7	190	1.83	5.64	1.28	0.956	668	93	0.00	0.020	0.90
1	8	0	200	1.26	-51.2	29.3	1192	819	47.0	22.9	0.77	0.18	1.50	0.62	8.7	17.3	204	1.66	6.07	>1.41	1.299	481	69	0.00	0.030	0.99
2	9	0	200	5.06	-56.2	29.8	1399	854	50.5	21.3	0.80	0.12	1.52	0.54	9.9	16.8	190	1.84	5.87	1.41	1.066	307	231	0.00	0.045	0.96
2	30	170	50	0.25	6.6	12.0	272	33	19.1	1.6	0.93	0.04	1.13	0.09	3.7	10.8	48	2.06	1.98	>1.60	0.685	129	33	-0.01	0.163	1.00
2	16	170	50	1.26	4.0	7.9	181	24	15.4	1.2	0.93	0.05	1.12	0.11	4.8	11.4	26	2.04	1.93	1.37	0.628	92	23	-0.01	0.146	1.00
4	13	170	50	5.06	-0.4	6.6	156	18	14.3	1.1	0.93	0.05	1.12	0.08	3.0	8.9	22	2.05	1.83	1.49	0.623	30	6	0.00	0.131	0.86
1	23	170	100	0.25	-4.8	23.1	512	167	28.0	6.4	0.87	0.08	1.25	0.29	4.2	13.9	124	1.97	3.24	0.81	0.731	648	138	0.01	0.044	0.97
1	1	170	100	1.26	-0.4	19.3	417	79	25.1	3.1	0.89	0.05	1.23	0.19	3.9	11.9	132	2.04	2.52	1.46	0.819	381	54	0.00	0.067	0.99
1	5	170	100	5.06	-1.0	15.7	369	84	22.9	3.6	0.90	0.06	1.16	0.17	4.8	14.3	94	1.99	2.48	1.45	0.865	97	18	0.00	0.080	0.94
1	10	170	200	0.25	-50.9	31.5	1250	852	49.1	23.6	0.76	0.17	1.54	0.56	9.7	16.3	97	1.94	3.32	1.18	0.851	1141	233	0.00	0.116	0.91
3	29	170	200	1.26	-52.5	33.0	1303	852	49.6	22.2	0.77	0.15	1.53	0.50	9.3	16.1	172	1.70	6.07	>1.38	0.985	474	94	0.00	0.044	0.99
2	12	170	200	5.06	-19.0	29.7	798	459	37.0	14.2	0.82	0.13	1.38	0.42	5.6	15.1	158	1.64	3.61	1.20	0.800	323	84	0.00	0.030	0.98
4	22	170	100	1.26	-6.2	20.3	469	137	26.3	5.0	0.89	0.07	1.21	0.23	4.3	13.7	110	2.06	2.73	1.30	0.875	304	60	0.00	0.052	0.99
2	15	170	100	1.26	-1.1	19.5	451	113	25.7	5.0	0.89	0.07	1.20	0.24	4.3	13.8	111	2.08	2.64	1.35	0.908	196	45	0.00	0.080	0.99
3	27	170	100	1.26	-22.4	18.6	521	184	27.7	6.8	0.89	0.07	1.22	0.28	4.7	13.3	107	1.77	2.67	1.38	0.820	228	48	0.00	0.076	0.99
1	24	340	50	0.25	2.0	8.2	182	22	15.6	1.1	0.92	0.05	1.13	0.10	3.2	9.8	27	2.09	1.75	1.19	0.617	60	14	-0.01	0.211	1.00
1	26	340	50	1.26	1.0	6.0	130	17	12.9	1.0	0.94	0.06	1.11	0.07	3.2	11.2	21	2.12	1.63	1.12	0.630	14	8	-0.04	0.336	0.93
2	3	340	50	5.06	4.3	6.0	144	19	13.5	1.2	0.94	0.06	1.10	0.08	4.9	11.1	17	2.02	1.96	>1.57	0.611	20	5	0.00	0.177	0.91
4	11	340	100	0.25	-4.2	15.5	352	49	22.5	2.4	0.91	0.04	1.19	0.15	3.0	10.4	85	2.02	2.14	1.35	0.807	141	33	-0.01	0.133	1.00
1	2	340	100	1.26	-0.7	13.0	291	34	20.1	1.7	0.91	0.04	1.15	0.11	3.6	10.3	59	2.15	1.71	0.90	0.650	74	16	0.00	0.177	0.99
3	6	340	100	5.06	-0.1	9.1	211	23	16.6	1.1	0.93	0.04	1.10	0.07	3.4	9.2	46	2.03	1.87	1.14	0.613	16	4	0.01	0.180	0.63
3	4	340	200	0.25	-32.2	33.2	1026	827	41.0	20.4	0.82	0.15	1.35	0.43	6.6	15.0	205	1.64	6.04	>1.28	1.137	657	112	0.00	0.040	0.94
2	19	340	200	1.26	-13.6	31.1	775	435	35.6	13.5	0.83	0.13	1.32	0.38	4.9	14.0	156	1.92	4.33	1.13	1.003	390	62	0.00	0.053	0.99
3	25	340	200	5.06	-45.1	29.2	1037	746	42.2	21.1	0.79	0.17	1.40	0.44	8.5	16.4	142	1.70	4.49	1.24	0.724	197	34	0.00	0.075	1.00

Comment: The DIA was perfomed on SEM-images aquired at Vacc = 5 kV and 200k magnification. $\Delta N/N_0$ and φ are the relative change in counted NPs and the fractional area coverage of NPs respectively. $\bar{X}_{Area}$, $\bar{X}_{Max FD}$, $\bar{X}_{FF}$, $\bar{X}_{Ellips.}$ and $\bar{X}_d$ are the mean NP cross-sectional area, max Ferret diameter, fill factor and center-to-center interparticle distance respectively while s_{Area}, $s_{FD max}$, s_{FF}, $s_{Ellips.}$ and s_d are the corresponding standard deviations. s_θ is the standard deviation of the discrepancy between the angle spanned by the centroids of the two nearest neighbours and that of a perfect hexagonal geometry. t_{eff}, E_{LSPR}, $\varepsilon_{2,LSPR}$ and *FWHM* are the modelled effective medium thickness, LSPR peak position, magnitude and width respectively. ***MSE*** is the mean square error of the SE model. t_0, τ, *A* and *K* are the parameters of the function fitted to $\Delta I/I_0$. **Adj.** R^2 is a goodness of fit measure.

Table S3: Summary of Data Extracted at IP1 and IP3 for the DoE Samples

Design of Experiment					Inspection Point 1 (IP1) SEM + Digital Image Analysis												Inspection Point 3 (IP3) SEM + Digital Image Analysis											
Wafer	Sample Run	$c_{Citrate}$ [µM]	c_{HAuCl4} [µM]	c_{H2O2} [M]	$\Delta N/N_0$	φ	$\bar{X}_{Area}$ [nm^2]	s_{Area} [nm^2]	$\bar{X}_{Max\ FD}$ [nm]	$s_{Max\ FD}$ [nm]	$\bar{X}_{FF}$	s_{FF}	$\bar{X}_{Ellip.}$	$s_{Ellip.}$	s_d [nm]	s_θ [deg.]	$\Delta N/N_0$	φ	$\bar{X}_{Area}$ [nm^2]	s_{Area} [nm^2]	$\bar{X}_{Max\ FD}$ [nm]	$s_{Max\ FD}$ [nm]	$\bar{X}_{FF}$	s_{FF}	$\bar{X}_{Ellip.}$	$s_{Ellip.}$	s_d [nm]	s_θ [deg.]
3	14	0	50	0,25	-0,32	0,263	853	487	38	15,4	0,806	0,143	1,40	0,46	6,72	16,25	-0,01	0,112	252	48	19	2,3	0,907	0,053	1,15	0,17	4,2	13,0
4	17	0	50	1,26	-0,12	0,187	461	187	27	7,7	0,870	0,090	1,26	0,33	4,5	15,0	-0,02	0,099	232	42	18	2,0	0,915	0,053	1,16	0,15	4,3	11,6
4	7	0	50	5,06	-0,18	0,205	533	265	29	10,1	0,857	0,107	1,28	0,36	5,5	16,2	-0,02	0,081	188	33	16	1,7	0,918	0,053	1,15	0,13	3,8	10,2
3	28	0	100	0,25	-0,59	0,296	1387	795	52	21,1	0,766	0,150	1,59	0,59	10,1	16,6	-0,13	0,149	367	105	23	5,3	0,886	0,076	1,23	0,35	5,5	15,7
4	18	0	100	1,26	-0,64	0,354	1850	1254	61	28,1	0,712	0,174	1,62	0,55	11,8	17,4	-0,05	0,154	360	107	24	5,2	0,873	0,080	1,31	0,35	5,0	14,9
4	20	0	100	5,06	-0,47	0,304	1140	725	47	20,9	0,761	0,160	1,54	0,58	8,7	16,6	-0,07	0,173	404	132	25	6,4	0,876	0,083	1,29	0,39	4,4	14,4
3	21	0	200	0,25	-0,86	0,452	5425	3762	112	51,6	0,639	0,192	1,78	0,56	21,1	16,7	-0,23	0,198	543	249	30	11,6	0,866	0,105	1,35	0,62	6,2	16,0
1	8	0	200	1,26	-0,78	0,425	3385	2607	87	43,3	0,650	0,204	1,78	0,60	12,3	17,4	-0,43	0,304	1076	662	45	20,5	0,785	0,154	1,51	0,60	7,8	16,7
2	9	0	200	5,06	-0,85	0,438	4830	3272	105	53,2	0,656	0,187	1,74	0,56	17,6	17,0	-0,44	0,256	999	532	41	15,5	0,835	0,094	1,43	0,48	8,4	16,9
2	30	170	50	0,25	0,05	0,159	360	41	22	2,0	0,918	0,042	1,15	0,12	4,1	13,1	0,06	0,073	176	16	15	0,9	0,930	0,050	1,13	0,06	3,2	9,5
2	16	170	50	1,26	0,01	0,120	278	32	20	1,7	0,923	0,045	1,15	0,12	4,4	11,5	0,05	0,062	144	19	13	1,0	0,936	0,055	1,09	0,06	3,8	10,2
4	13	170	50	5,06	-0,02	0,112	258	23	19	1,2	0,920	0,040	1,15	0,09	2,5	8,8	-0,02	0,064	149	17	14	0,9	0,936	0,055	1,11	0,06	2,9	9,7
1	23	170	100	0,25	-0,30	0,322	936	617	40	17,6	0,808	0,153	1,38	0,44	6,1	15,7	0,02	0,128	279	49	20	2,6	0,900	0,055	1,19	0,20	4,5	12,1
1	31	170	100	1,26	-0,11	0,305	727	300	33	9,1	0,872	0,094	1,26	0,28	3,9	13,1	0,00	0,126	282	45	20	2,2	0,904	0,049	1,20	0,19	3,9	10,3
1	5	170	100	5,06	-0,13	0,221	570	230	29	8,6	0,884	0,087	1,24	0,31	4,6	14,5	-0,04	0,142	331	84	22	4,1	0,899	0,067	1,16	0,20	5,2	16,0
1	10	170	200	0,25	-0,85	0,427	4957	4582	109	68,0	0,563	0,233	1,82	0,66	19,4	18,4	-0,25	0,226	644	282	33	10,9	0,832	0,109	1,39	0,47	6,1	15,7
3	29	170	200	1,26	-0,80	0,447	3636	3568	89	59,4	0,666	0,218	1,71	0,59	16,8	16,6	-0,40	0,265	897	575	40	18,0	0,790	0,149	1,49	0,51	8,2	16,5
2	12	170	200	5,06	-0,71	0,434	2677	2602	73	43,1	0,658	0,223	1,59	0,60	14,8	17,9	-0,16	0,301	788	429	37	13,5	0,816	0,135	1,38	0,44	5,3	15,2
4	22	170	100	1,26	-0,29	0,297	843	453	36	13,0	0,851	0,125	1,28	0,33	5,6	14,9	-0,01	0,126	292	55	20	2,8	0,905	0,060	1,14	0,18	4,8	13,1
2	15	170	100	1,26	-0,22	0,313	879	456	37	13,4	0,846	0,128	1,30	0,35	5,4	14,9	-0,06	0,147	359	66	23	2,9	0,898	0,051	1,16	0,16	5,5	14,4
3	27	170	100	1,26	-0,22	0,269	743	281	33	8,4	0,881	0,082	1,22	0,24	4,5	13,1	-0,20	0,118	323	98	22	4,7	0,899	0,073	1,20	0,27	5,7	13,2
1	24	340	50	0,25	-0,01	0,062	146	23	14	1,2	0,937	0,052	1,09	0,07	3,1	9,6	0,00	0,118	261	36	19	1,8	0,914	0,047	1,17	0,12	3,4	10,2
1	26	340	50	1,26	0,02	0,089	190	22	16	1,2	0,924	0,049	1,16	0,10	2,9	9,5	0,00	0,058	129	20	13	1,1	0,933	0,061	1,11	0,07	3,5	11,1
2	33	340	50	5,06	0,09	**0,044**	102	16	11	1,0	0,944	0,071	1,09	0,06	3,6	10,0	0,02	0,080	191	25	16	1,5	0,921	0,052	1,15	0,11	5,0	11,7
4	11	340	100	0,25	-0,08	0,272	634	143	31	4,8	0,885	0,050	1,21	0,15	3,3	10,2	-0,05	0,164	375	49	23	2,2	0,906	0,040	1,18	0,13	3,9	12,5
1	32	340	100	1,26	0,01	0,189	398	47	24	1,8	0,907	0,039	1,19	0,16	3,5	10,3	-0,01	0,084	193	25	16	1,3	0,928	0,048	1,13	0,11	3,4	11,3
3	6	340	100	5,06	-0,01	0,136	302	33	21	1,5	0,920	0,038	1,16	0,10	3,8	10,7	-0,02	0,089	214	23	17	1,2	0,931	0,046	1,11	0,08	2,7	9,1
3	34	340	200	0,25	-0,80	0,497	3957	3862	90	56,5	0,623	0,252	1,65	0,57	22,0	18,2	-0,03	0,219	491	180	27	6,6	0,882	0,073	1,23	0,31	4,2	13,4
2	19	340	200	1,26	-0,68	0,456	2570	2773	72	49,7	0,668	0,221	1,61	0,57	12,4	16,3	-0,17	0,264	698	367	33	12,0	0,828	0,127	1,29	0,34	5,5	15,5
3	25	340	200	5,06	-0,78	0,415	3288	2742	82	42,7	0,629	0,210	1,64	0,50	17,5	17,6	-0,08	0,156	369	109	23	4,6	0,900	0,063	1,15	0,18	5,0	14,7

Comment: The DIA was perfomed on SEM-images aquired at Vacc = 5 kV and 200k magnification. $\Delta N/N_0$ *and* φ are the relative change in counted NPs and the fractional area coverage of NPs respectively. $\bar{X}_{Area}$, $\bar{X}_{Max\ FD}$, $\bar{X}_{FF}$, $\bar{X}_{Ellips.}$ and $\bar{X}_d$ are the mean NP cross-sectional area, max Ferret diameter, fill factor and center-to-center interparticle distance respectively while s_{Area}, $s_{FD\ max}$, s_{FF}, $s_{Ellips.}$ and s_d are the corresponding standard deviations. s_θ is the standard deviation of the discrepancy between the angle spanned by the centroids of the two nearest neighbours and that of a perfect hexagonal geometry.

		$c_{citrate}$	c_{HAuCl4}	c_{H2O2}	$\Delta N/N_0$	φ	$\bar{X}_{Area}$	s_{Area}	$\bar{X}_{Max\ FD}$	$s_{Max\ FD}$	$\bar{X}_{FF}$	s_{FF}	$\bar{X}_{Ellips.}$	$s_{Ellips.}$	s_d	s_θ	t_{eff}	E_{LSPR}	$\varepsilon_{2,LSPR}$	t_0	τ	A	K
Reagent conc.	$c_{citrate}$	1																					
	c_{HAuCl4}	0.00	1																				
	c_{H2O2}	0.00	0.01	1																			
SEM + DIA	$\Delta N/N_0$	0.31	-0.85	0.04	1																		
	φ	-0.20	0.93	-0.15	-0.83	1																	
	$\bar{X}_{Area}$	-0.26	0.92	-0.08	-0.96	0.93	1																
	s_{Area}	-0.17	0.92	-0.05	-0.95	0.89	0.98	1															
	$\bar{X}_{Max\ FD}$	-0.30	0.92	-0.10	-0.95	0.95	0.99	0.96	1														
	$s_{Max\ FD}$	-0.22	0.92	-0.05	-0.96	0.92	0.98	0.99	0.98	1													
	$\bar{X}_{FF}$	0.29	-0.90	0.08	0.93	-0.94	-0.96	-0.95	-0.98	-0.98	1												
	s_{FF}	-0.17	0.88	-0.03	-0.89	0.89	0.90	0.94	0.91	0.96	-0.96	1											
	$\bar{X}_{Ellips}$	-0.40	0.86	-0.10	-0.95	0.90	0.96	0.92	0.97	0.95	-0.97	0.91	1										
	s_{Ellips}	-0.47	0.81	-0.10	-0.91	0.90	0.91	0.88	0.94	0.93	-0.95	0.91	0.97	1									
	s_d	-0.34	0.80	0.03	-0.95	0.78	0.94	0.93	0.92	0.93	-0.90	0.85	0.91	0.86	1								
	s_θ	-0.51	0.73	-0.03	-0.82	0.85	0.85	0.80	0.88	0.86	-0.89	0.85	0.89	0.93	0.84	1							
SE	t_{eff}	-0.29	0.86	-0.11	-0.77	0.92	0.86	0.82	0.88	0.83	-0.84	0.77	0.81	0.83	0.71	0.80	1						
	E_{LSPR}	0.34	-0.67	-0.08	0.73	-0.73	-0.70	-0.73	-0.73	-0.76	0.77	-0.80	-0.74	-0.79	-0.66	-0.75	-0.74	1					
	$\varepsilon_{2,LSPR}$	-0.27	0.86	-0.08	-0.88	0.88	0.93	0.93	0.92	0.92	-0.88	0.86	0.87	0.86	0.85	0.81	0.92	-0.77	1				
Unseeded Media	t_0	-0.37	0.56	-0.46	-0.63	0.72	0.68	0.65	0.71	0.68	-0.73	0.65	0.72	0.72	0.62	0.67	0.59	-0.45	0.57	1			
	τ	-0.46	0.51	-0.22	-0.66	0.66	0.70	0.64	0.71	0.66	-0.68	0.58	0.74	0.73	0.65	0.67	0.54	-0.42	0.56	0.80	1		
	A	-0.14	0.21	0.17	-0.09	0.26	0.16	0.11	0.19	0.12	-0.16	0.09	0.13	0.15	0.12	0.10	0.29	-0.21	0.19	0.20	0.09	1	
	K	0.64	-0.47	-0.01	0.49	-0.68	-0.55	-0.47	-0.61	-0.53	0.60	-0.51	-0.60	-0.68	-0.48	-0.71	-0.74	0.66	-0.61	-0.55	-0.50	-0.61	1
		Reagent conc.			SEM + DIA												SE			Unseeded Media			

1.00
0.75
0.50
0.25
0.00
-0.25
-0.50
-0.75
-1.00

Figure S6: (Pearson) Correlation matrix of reagent concentrations and the various parameters extracted from the DoE samples (IP2) and the unseeded growth media.

Table S4: Summary of Extracted Parameters Modelled Using Multiple Linear Regression (MLR)

**Method**	Backwards Elimination (α = 0.05)
**Variables**	$c_{citrate}$ ("c_{cit}"), c_{HAuCl4} ("c_{Au}"), c_{H2O2} ("c_{HP}") + Interaction and 2nd order terms
**Units**	[c_{cit}] = µM. [c_{Au}] = µM. [c_{HP}] = M

			Goodness of Fit			R^2 Contribution						
	f(x)	***Full Expression***	R^2	*Adj.* R^2	R^2_{pred}	c_{cit}	c_{Au}	c_{HP}	$c_{cit}c_{Au}$	$c_{cit}c_{HP}$	$c_{Au}c_{HP}$	c_{Au}^2
**SEM + DIA**	**$\Delta N/N_0$**	$f = 0.0950 + 0.000475\ c_{cit} - 0.002892\ c_{Au}$	81.1%	79.7%	76.4%	(+) 9.5%	(-) 71.6%	-	-	-	-	-
	φ	$f = 0.1079 - 0.000345\ c_{cit} + 0.001075\ c_{Au} - 0.00705\ c_{HP} + 0.000002\ c_{cit}c_{Au}$	95.0%	94.2%	92.9%	(-) 3.9%	(+) 85.7%	(-) 2.5%	(+) 2.8%	-	-	-
	$\bar{x}_{Area}$ [nm²]	$f = 10.6 - 0.769\ c_{cit} + 6.071\ c_{Au}$	92.1%	91.5%	90.0%	(-) 6.7%	(+) 85.3%	-	-	-	-	-
	s_{Area} /[nm²]	$f = 138 - 0.397\ c_{cit} - 1.97\ c_{Au} + 0.02632\ c_{Au}^2$	91.7%	90.7%	88.8%	(-) 2.8%	(-) 84.8%	-	-	-	-	(+) 4.1%
	$\bar{x}_{Max\ FD}$ [nm]	$f = 13.50 - 0.02646\ c_{cit} + 0.18332\ c_{Au} - 0.660\ c_{HP}$	95.2%	94.6%	93.1%	(-) 8.8%	(+) 85.2%	(-) 1.2%	-	-	-	-
	$s_{Max\ FD}$ [nm]	$f = 2.81 - 0.01321\ c_{cit} + 0.0016\ c_{Au} + 0.000476\ c_{Au}^2$	91.9%	90.9%	89.1%	(-) 4.7%	(+) 85.1%	-	-	-	-	(+) 2.0%
	$\bar{x}_{FF}$	$f = 0.94535 + 0.000122\ c_{cit} - 0.000839\ c_{Au}$	89.9%	89.1%	87.1%	(+) 8.5%	(-) 81.3%	-	-	-	-	-
	s_{FF}	$f = 0.01438 + 0.000645\ c_{Au}$	77.2%	76.4%	74.3%	-	(+) 77.2%	-	-	-	-	-
	$\bar{x}_{Ellip}$	$f = 1.1058 - 0.000441\ c_{cit} + 0.002094\ c_{Au}$	89.1%	88.2%	86.5%	(-) 15.9%	(+) 73.1%	-	-	-	-	-
	s_{Ellip}	$f = 0.1196 - 0.000630\ c_{cit} + 0.002425\ c_{Au}$	86.9%	85.9%	83.9%	(-) 21.7%	(+) 65.2%	-	-	-	-	-
	s_d [nm]	$f = 3.353 - 0.00011\ c_{cit} + 0.00956\ c_{Au} + 0.000106\ c_{Au}^2 - 0.000043\ c_{cit}c_{Au}$	83.8%	81.8%	76.6%	(-) 0.2%	(+) 79.6%	-	(-) 2.6%	-	-	(+) 1.3%
	s_θ [nm]	$f = 9.646 - 0.00927\ c_{cit} + 0.0661\ c_{Au} - 0.000134\ c_{Au}^2$	84.2%	82.8%	80.2%	(-) 0.3%	(+) 79.7%	-	-	-	-	(-) 4.2%
										-		
**SE**	t_{eff} [Å]	$f = 1.67 - 0.1248\ c_{cit} + 1.415\ c_{Au} - 0.002374\ c_{Au}^2$	90.8%	90.0%	88.3%	(-) 1.8%	(+) 86.6%	-	-	-	-	(-) 2.4%
	E_{LSPR} [eV]	$f = 2.1377 + 0.000272\ c_{cit} - 0.002206\ c_{Au}$	68.5%	66.6%	62.5%	(+) 0.8%	(-) 67.7%	-	-	-	-	-
	$\varepsilon_{2,LSPR}$ [ε_0]	$f = 0.422 - 0.002225\ c_{cit} + 0.02720\ c_{Au} + 0.1972\ c_{HP} - 0.001910\ c_{Au}c_{HP}$	89.2%	87.9%	84.1%	(-) 3.6%	(+) 83.7%	(+) 0.0%	-	-	(-) 1.9%	-
	FWHM[b] [eV]	$f = 0.4752 + 0.01352\ c_{Au} - 0.000049\ c_{Au}^2$	76.9%	75.4%	73.6%	-	(+) 42,9%	-	-	-	-	(-) 34.0%
**Extinction**	$\ln(t_0)$	$f = 6.280 - 0.01013\ c_{cit} + 0.00273\ c_{Au} - 0.2471\ c_{HP} + 0.000047\ c_{cit}c_{Au}$	89.1%	87.4%	84.7%	(-) 28.3%	(+) 30.8%	(-) 18.5%	(+) 11.6%	-	-	-
	$\ln(\tau)$	$f = 4.558 - 0.00660\ c_{cit} + 0.00148\ c_{Au} - 0.2536\ c_{HP} + 0.000030\ c_{cit}c_{Au} - 0.000707\ c_{cit}c_{HP} + 0.001467\ c_{Au}c_{HP}$	87.0%	83.6%	73.8%	(-) 31.9%	(+) 29.5%	(-) 14.2%	(+) 5.5%	(-) 3.3%	(+) 2.8%	-
	K	$f = 0.1146 + 0.000740\ c_{cit} - 0.001670\ c_{Au} + 0.000006\ c_{Au}^2 - 0.000003\ c_{cit}c_{Au}$	83.1%	80.4%	74.2%	(+) 41.1%	(-) 22.5%	-	(-) 14.8%	-	-	(+) 4.7%

Comment: [a]R^2 - contribution of the significant linear ($c_{citrate}$, c_{HAuCl4} and c_{H2O2}), interaction ($c_{citrate}\cdot c_{HAuCl4}$, $c_{citrate}\cdot c_{H2O2}$ and $c_{HAuCl4}\cdot c_{H2O2}$) and square (c_{HAuCl4}^2) terms to the total R^2-value of the models. The contributions from all significant terms add up to the total R^2-value of the model. The sign of the coefficients are stated within the parenthesis. The reference samples are included in the modeling of s_d, s_θ and the SE features. [b]Data points for which FWHM extends beyond the measured spectral range are not included.

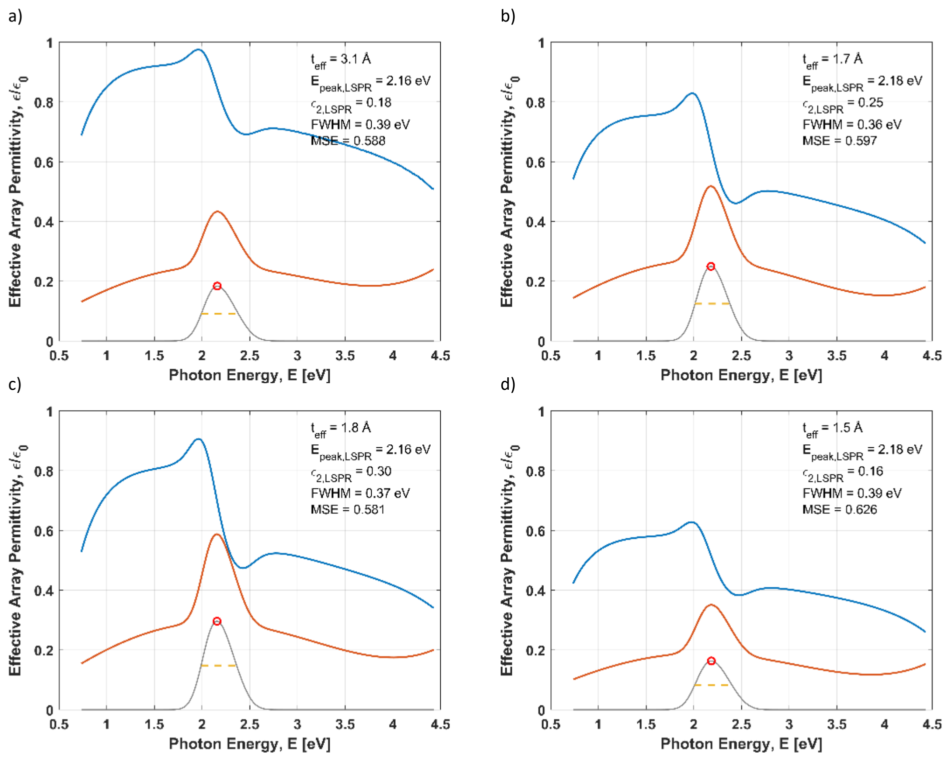

Figure S7: SE-modelled relative permittivity (ϵ) and thickness (t_{eff}) of the effective medium formed by the NP arrays (at IP2) on pre-SMNPG reference samples taken from parent wafer a) 1, b) 2, c) 3 and d) 4. Line color legend: blue $= \epsilon_1(\lambda)$, orange $= \epsilon_2(\lambda)$, gray $= \epsilon_2$ contribution of oscillators corresponding to the LSPR peak, dashed line $= FWHM$.

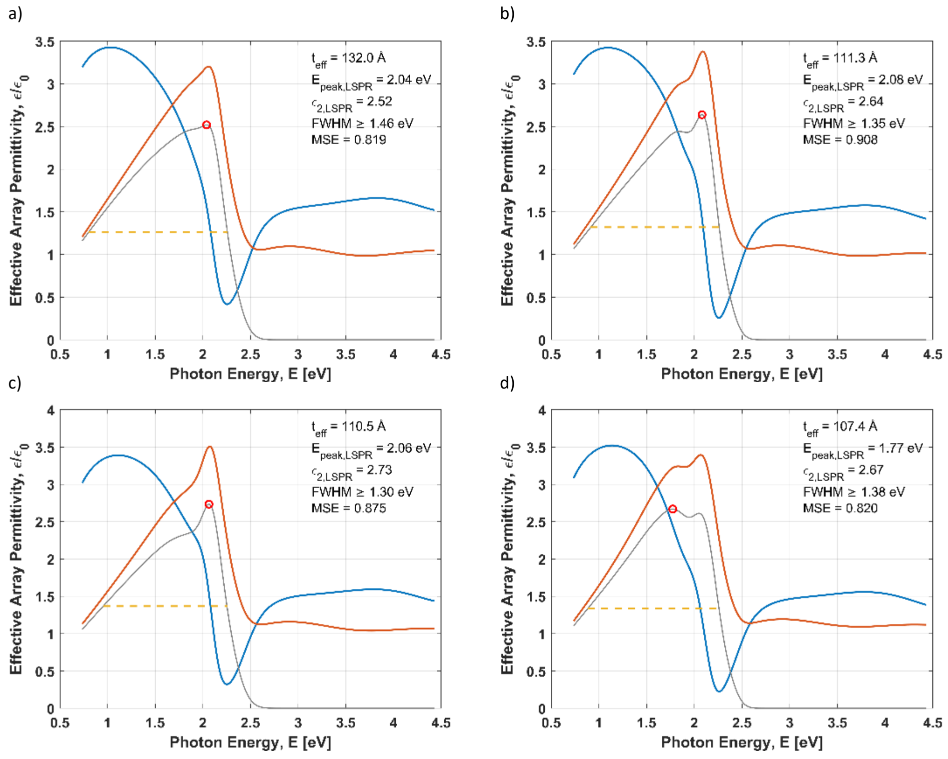

Figure S8: SE-modelled relative permittivity (ϵ) and thickness (t_{eff}) of the effective medium formed by the NP arrays (at IP2) on the DoE centerpoint replicates i.e. run a) 1, b) 15, c) 22 and d) 27. Line color legend: blue $= \epsilon_1(\lambda)$, orange $= \epsilon_2(\lambda)$, gray $= \epsilon_2$ contribution of oscillators corresponding to the LSPR peak, dashed line $= FWHM$.

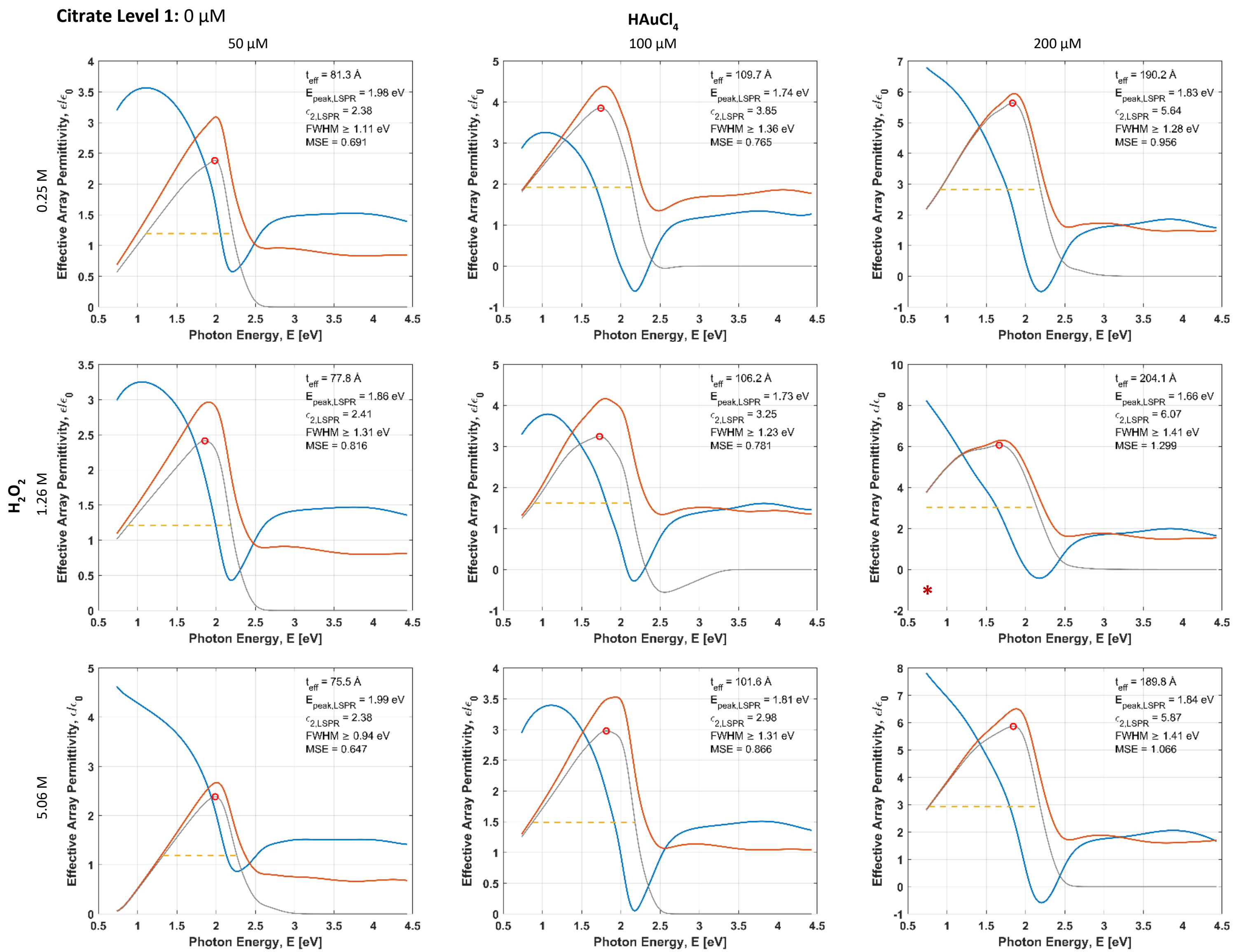

*Figure S9: SE-modelled relative permittivity (ϵ) and thickness (t_{eff}) of the effective medium formed by the NP arrays (at IP2) on DoE samples with citrate level 1 ($c_{citrate} = 0\ \mu M$). Line color legend: blue $= \epsilon_1(\lambda)$, orange $= \epsilon_2(\lambda)$, gray = ϵ_2 contribution of oscillators corresponding to the LSPR peak, dashed line $= FWHM$. *FWHM-value could not be ascertained.*

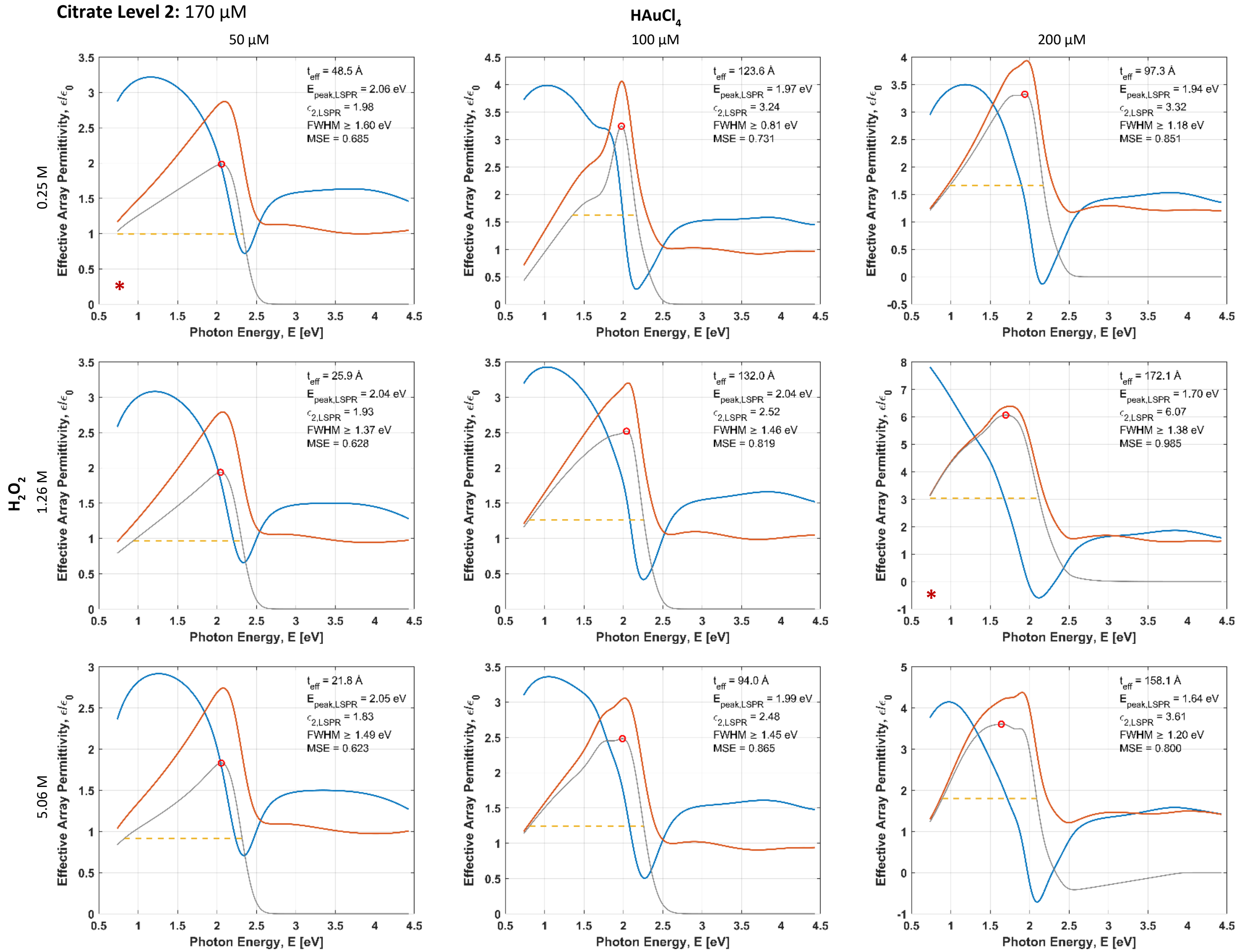

*Figure S10: SE-modelled relative permittivity (ϵ) and thickness (t_{eff}) of the effective medium formed by the NP arrays (at IP2) on DoE samples with citrate level 2 ($c_{citrate} = 170\ \mu M$). Line color legend: blue $= \epsilon_1(\lambda)$, orange $= \epsilon_2(\lambda)$, gray = ϵ_2 contribution of oscillators corresponding to the LSPR peak, dashed line $= FWHM$.*FWHM-value could not be ascertained.*

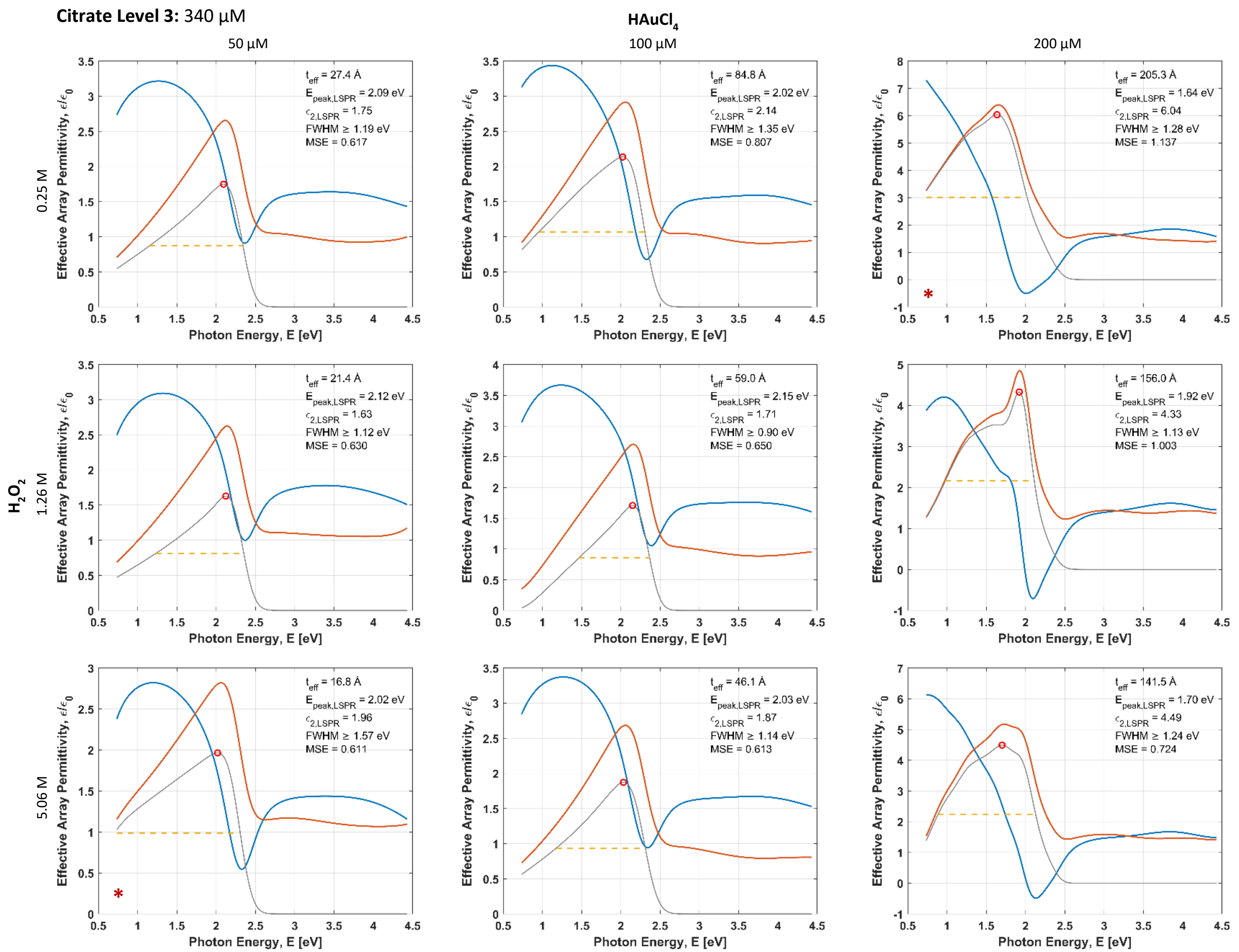

*Figure S11: SE-modelled relative permittivity (ϵ) and thickness (t_{eff}) of the effective medium formed by the NP arrays (at IP2) on DoE samples with citrate level 3 ($c_{citrate} = 340\ \mu M$). Line color legend: blue $= \epsilon_1(\lambda)$, orange $= \epsilon_2(\lambda)$, gray = ϵ_2 contribution of oscillators corresponding to the LSPR peak, dashed line $= FWHM$.*FWHM-value could not be ascertained.*

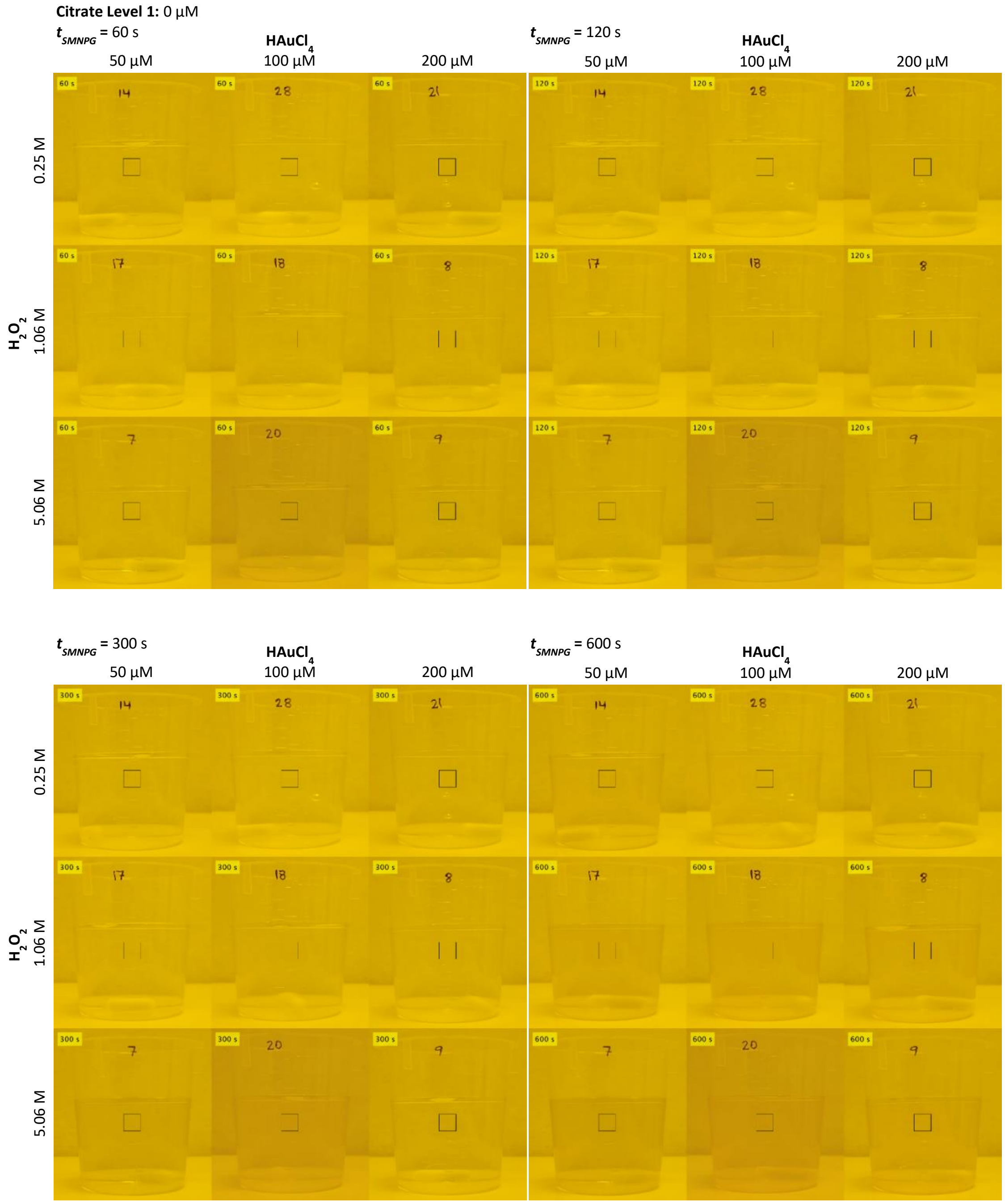

Figure S12: Compilations of extracted video frames of the unseeded DoE growth media with citrate level 1 ($c_{citrate} = 0\ \mu M$) after 1, 2, 5 and 10 min. DoE run order written on the beakers. The difference in background color for run 20 (i.e. $c_{HAuCl4} = 100\ \mu M, c_{H2O2} = 5.06\ M$) is a side-effect of concatenating two movie files but should not affect the $\Delta I(t)/I_0$ due to the run-specific internal normalization.

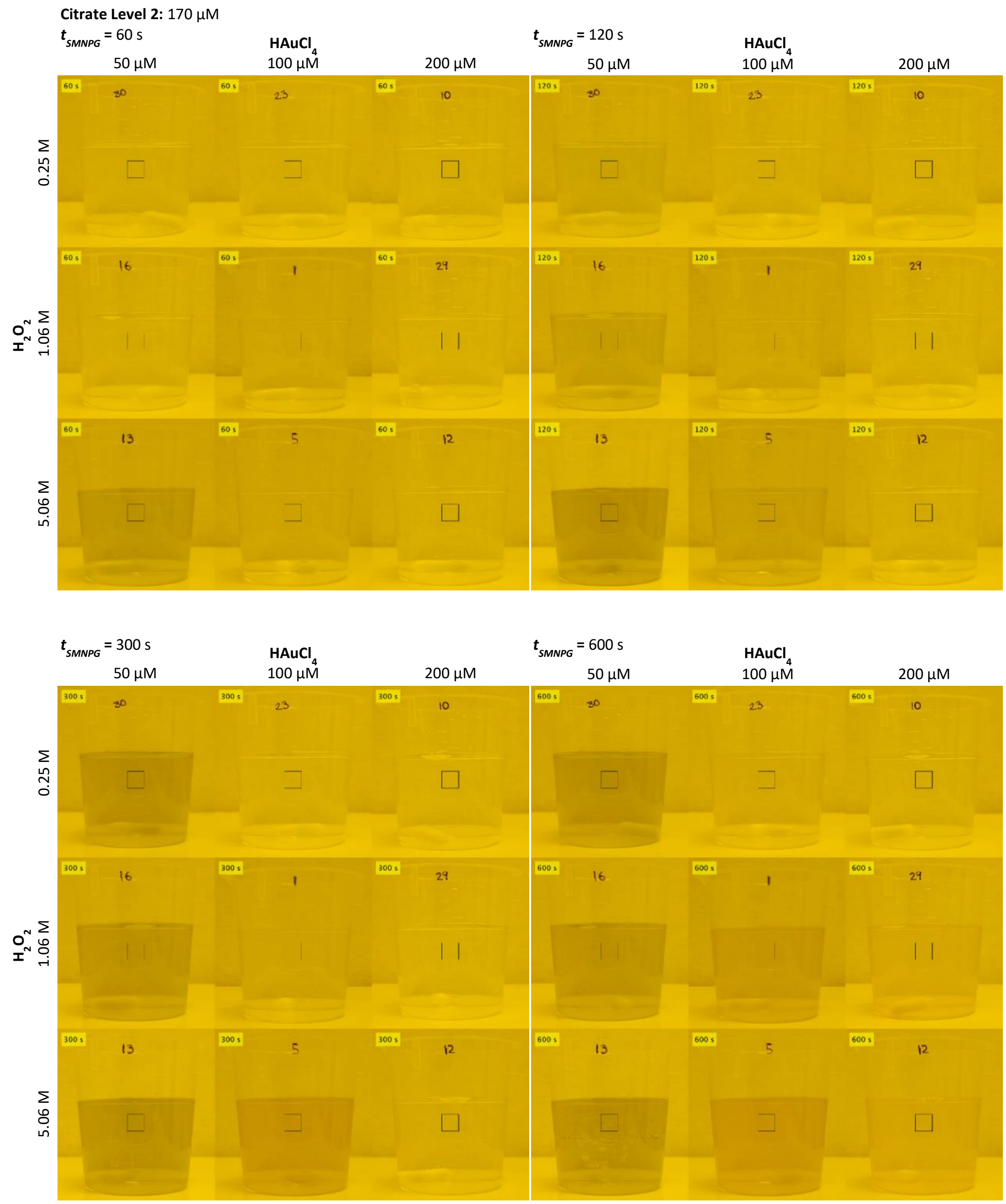

Figure S13: Compilations of extracted video frames of the unseeded DoE growth media with citrate level 2 ($c_{citrate} = 170\ \mu M$) after 1, 2, 5 and 10 min. DoE run order written on the beakers.

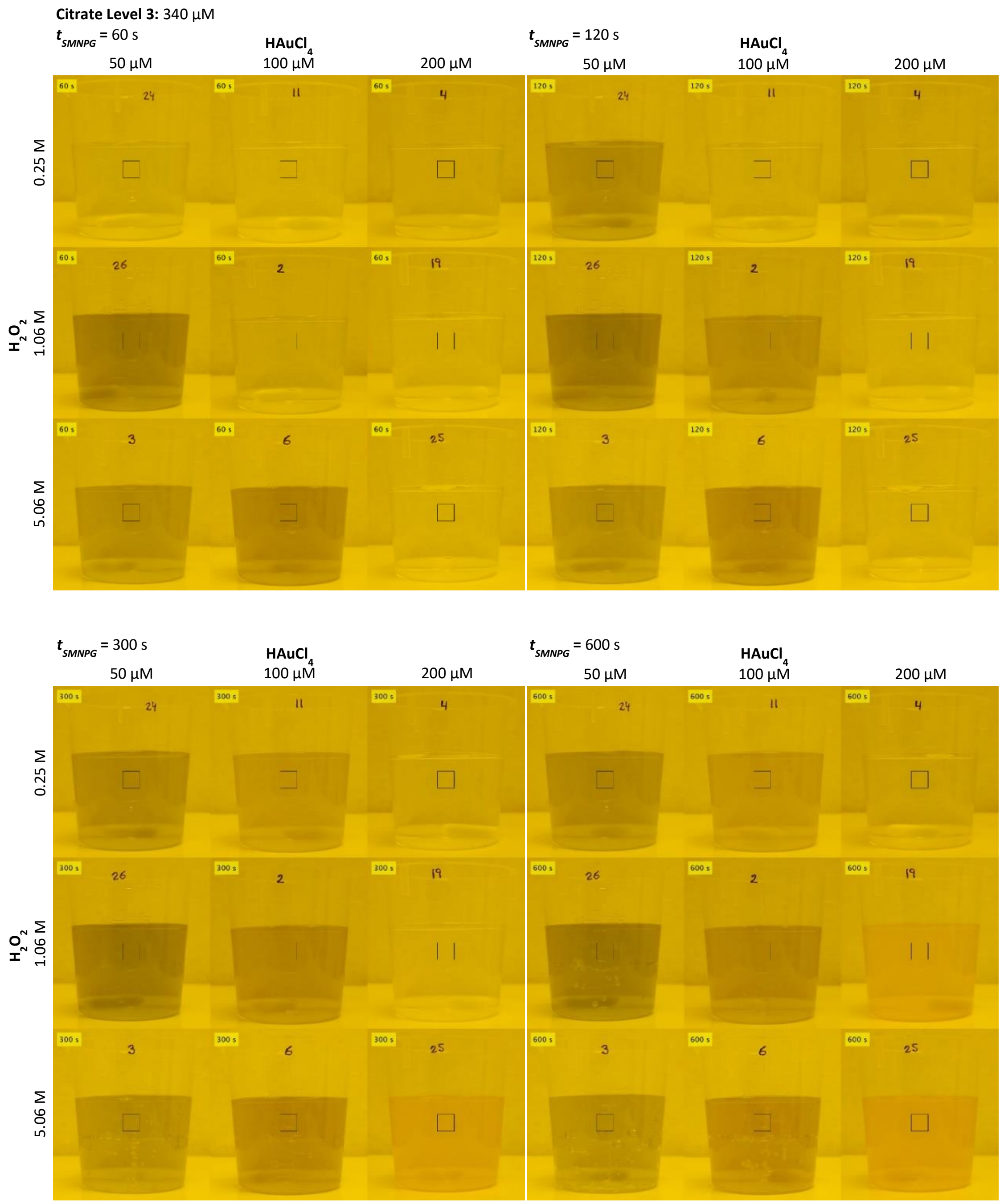

Figure S14: Compilations of extracted video frames of the unseeded DoE growth media with citrate level 2 ($c_{citrate} = 170\ \mu M$) after 1, 2, 5 and 10 min. DoE run order written on the beakers.

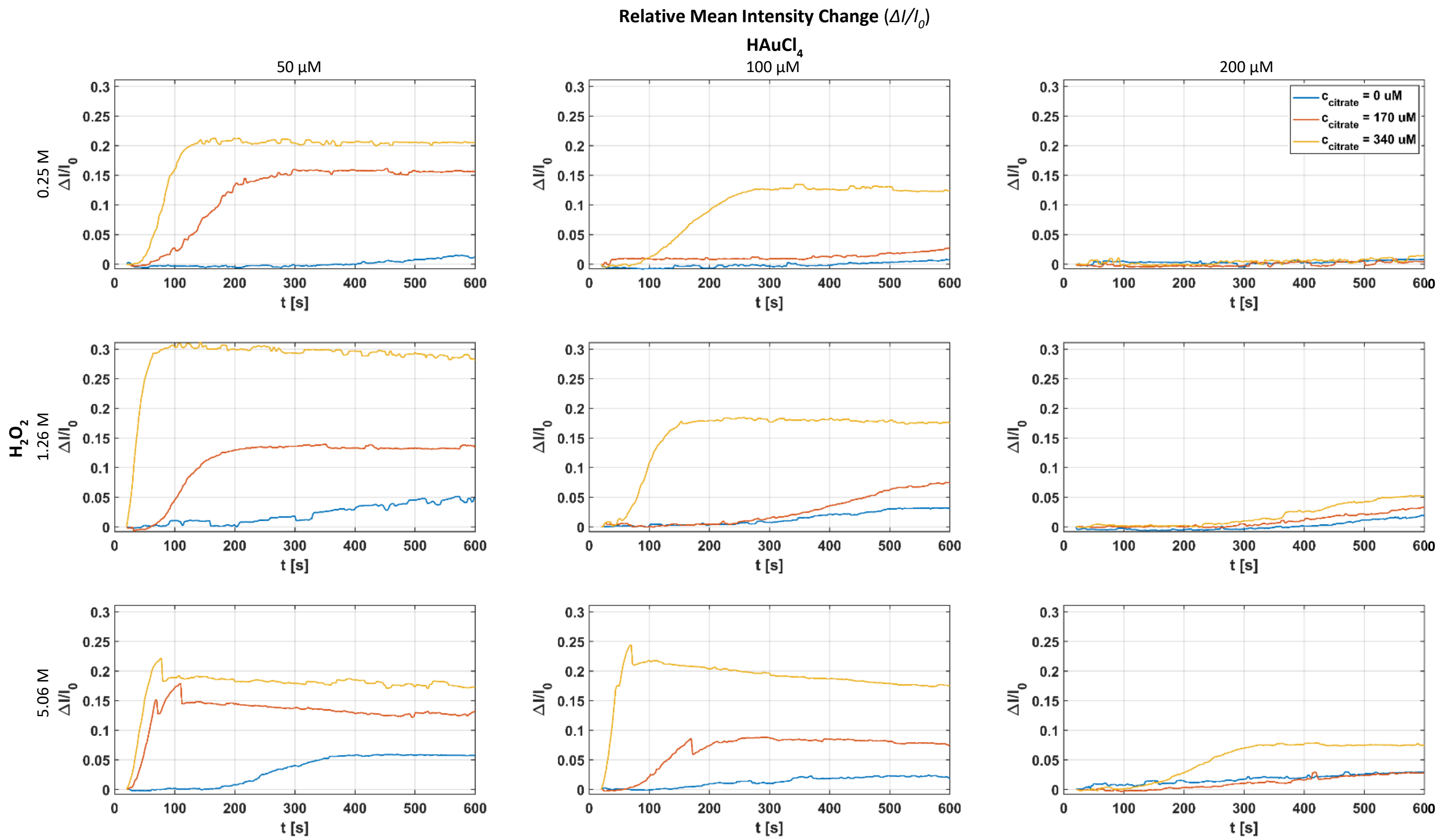

Figure S15: Compilation of the normalized differential mean pixel intensity ($\Delta I/I_0$) *data of the DoE unseeded media. A 3-datapoint sweeping median filter has been used for noise reduction.*

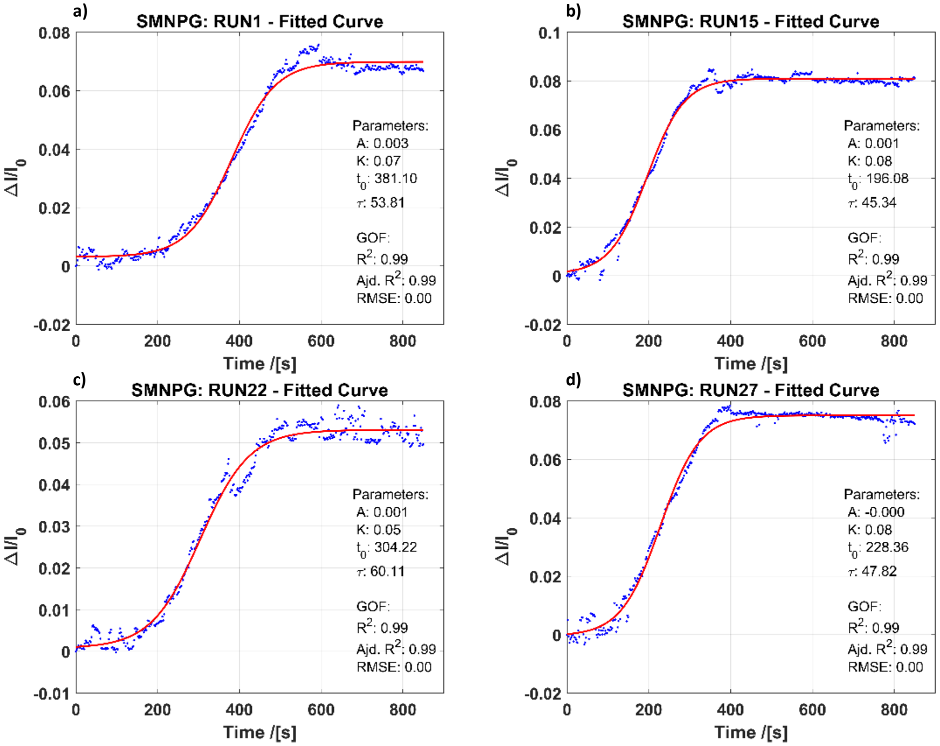

Figure S16: Normalized differential mean pixel intensity ($\Delta I/I_0$) data and associated curve fits for DoE centerpoint replicates, i.e. runs a) 1, b) 15, c) 22 and d) 27, for the unseeded growth media.

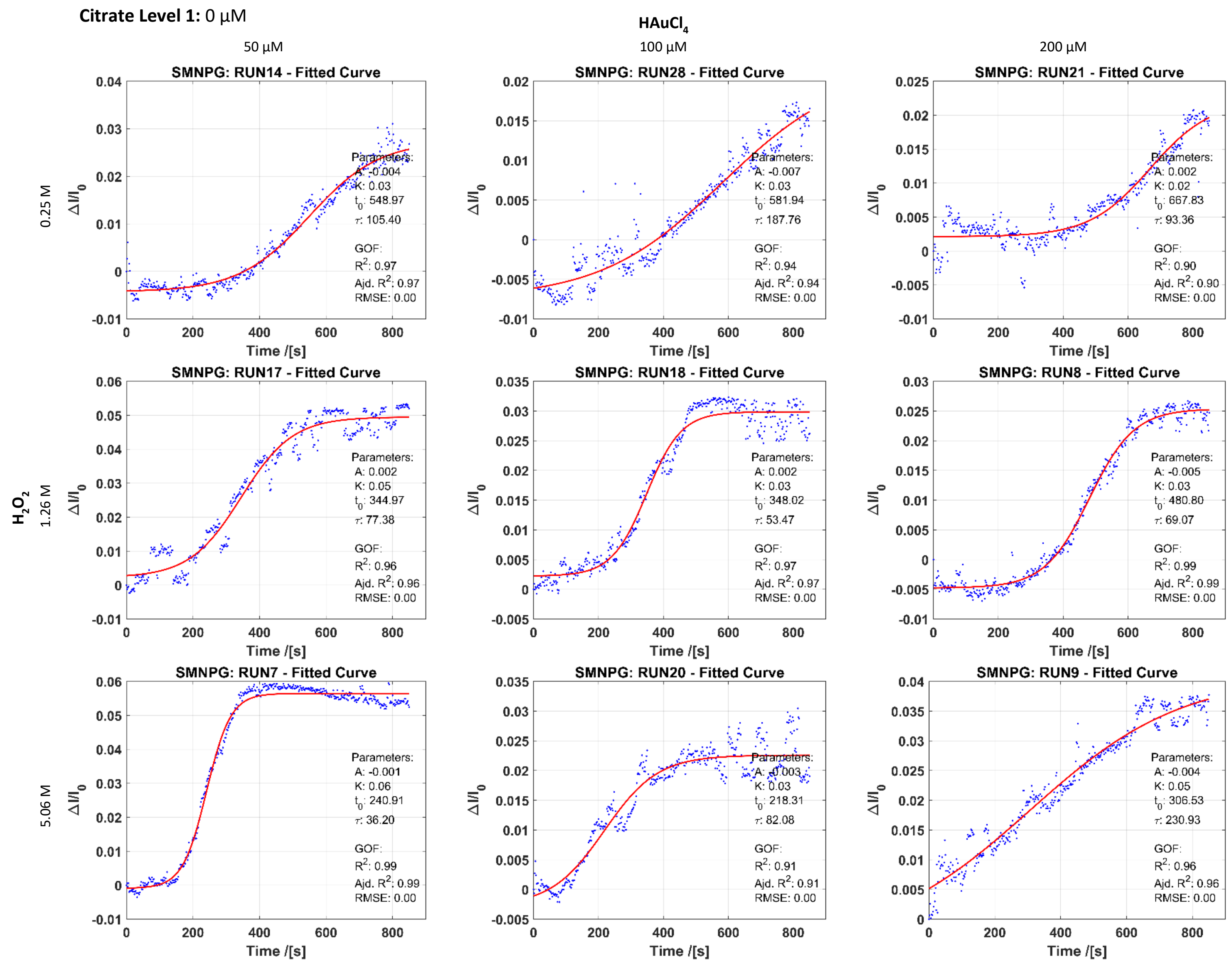

Figure S17: Normalized differential mean pixel intensity ($\Delta I/I_0$) data and curve fits for DoE unseeded media with citrate level 1 ($c_{citrate} = 0\ \mu M$).

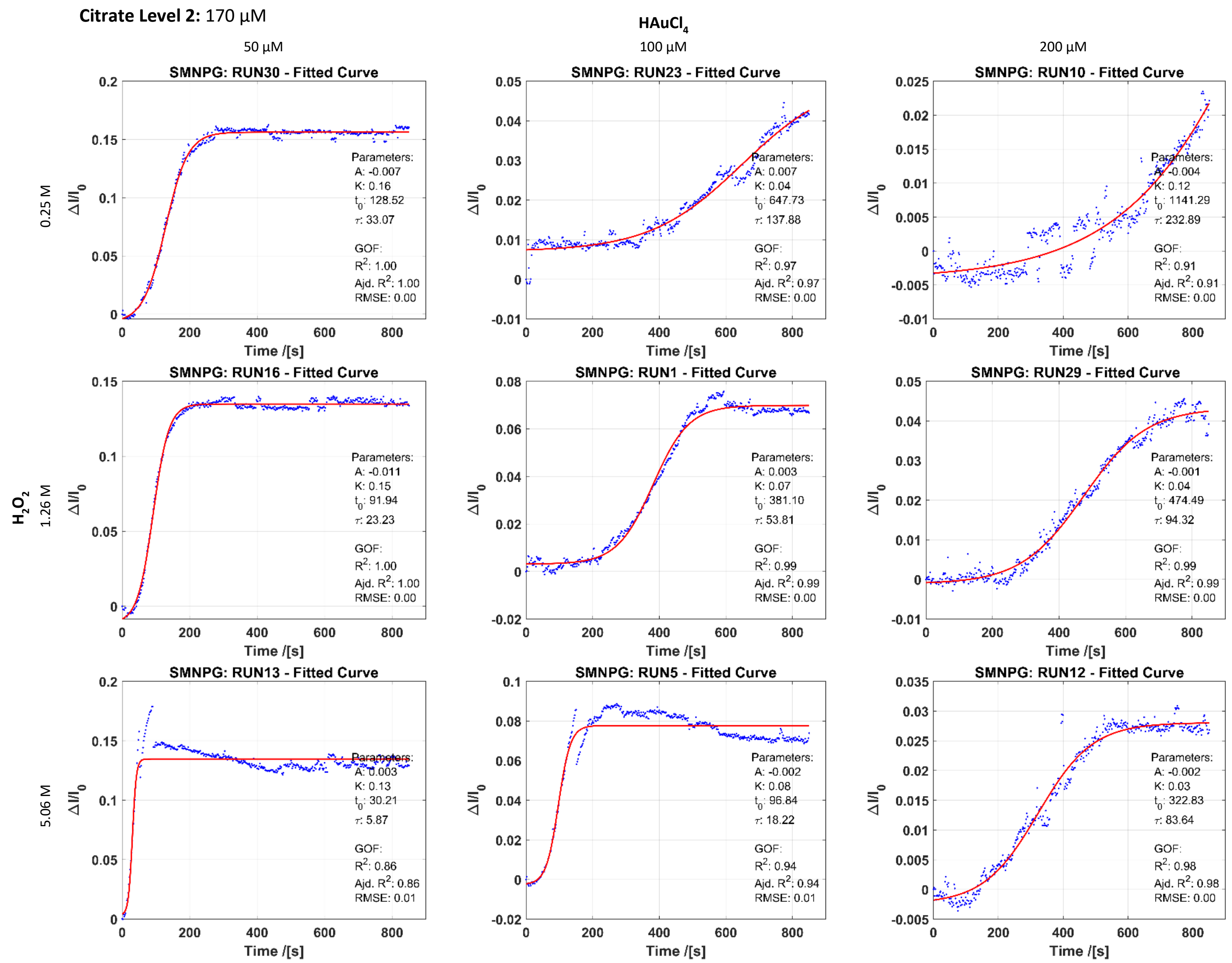

Figure S18: Normalized differential mean pixel intensity ($\Delta I/I_0$) data and curve fits for DoE unseeded media with citrate level 2 ($c_{citrate} = 170\ \mu M$).

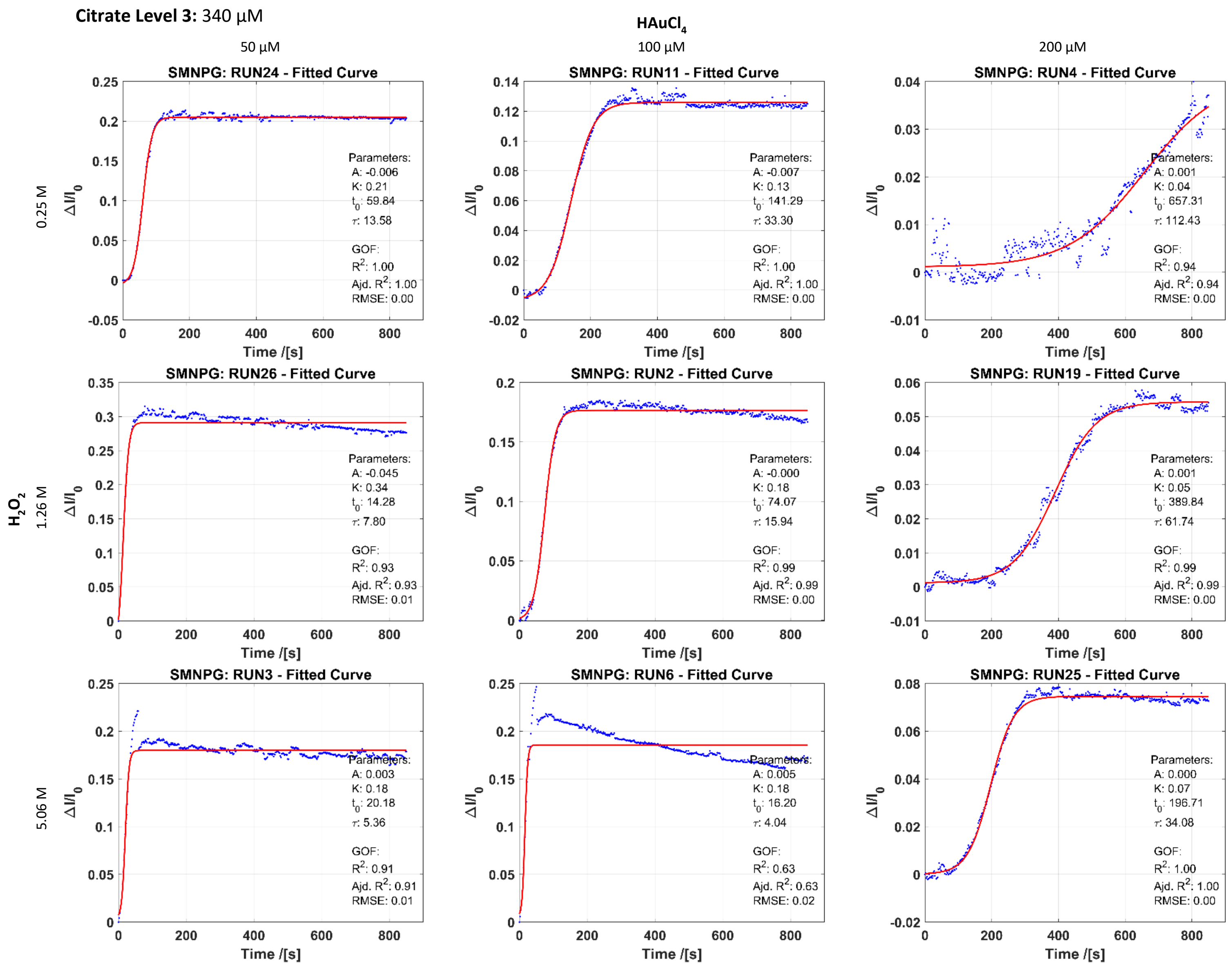

Figure S19: Normalized differential mean pixel intensity ($\Delta I/I_0$) data and curve fits for DoE unseeded media with citrate level 3 ($c_{citrate} = 340\ \mu M$).

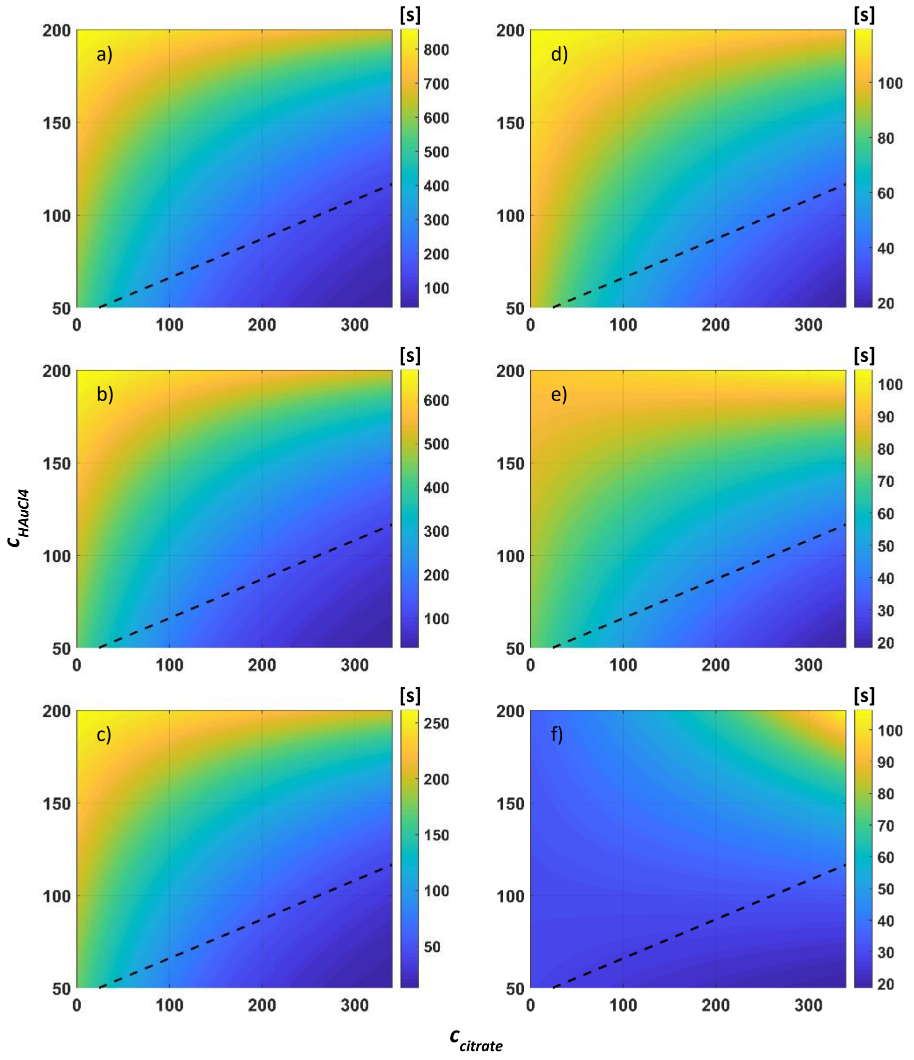

Figure S20: Heatmaps of t_0 (a-c) and τ (d-f) plotted for the three c_{H2O2} DoE-levels: 0.25 M (a, d), 1.26 M (b, e) and 5.06 M (c, f).

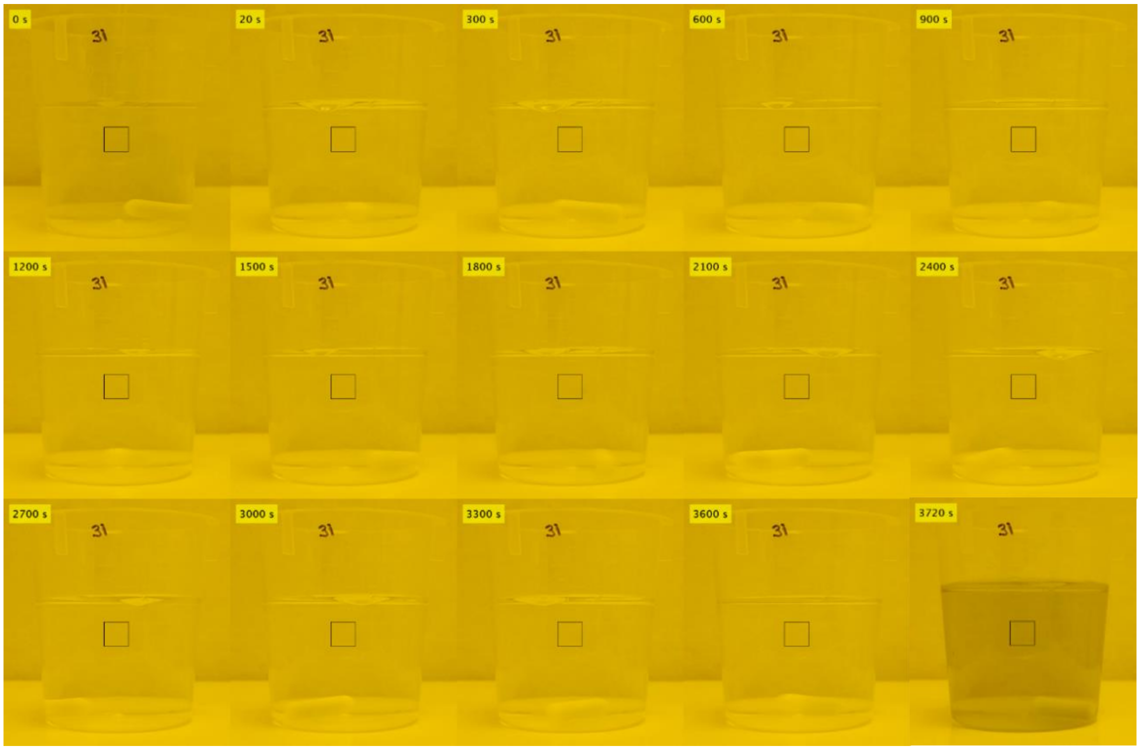

Figure S21: Supplementary run of an unseeded growth medium with the highest citrate ($c_{citrate} = 340\ \mu M$) and lowest $HAuCl_4$ ($c_{HAuCl4} = 50\ \mu M$) DoE levels, maintaining the total volume of 40 mL while not including any H_2O_2. Ran for 60 min without change in medium appearance, upon which 5 mL 31% H_2O_2 was added and the appearance changed rapidly (<2 min).

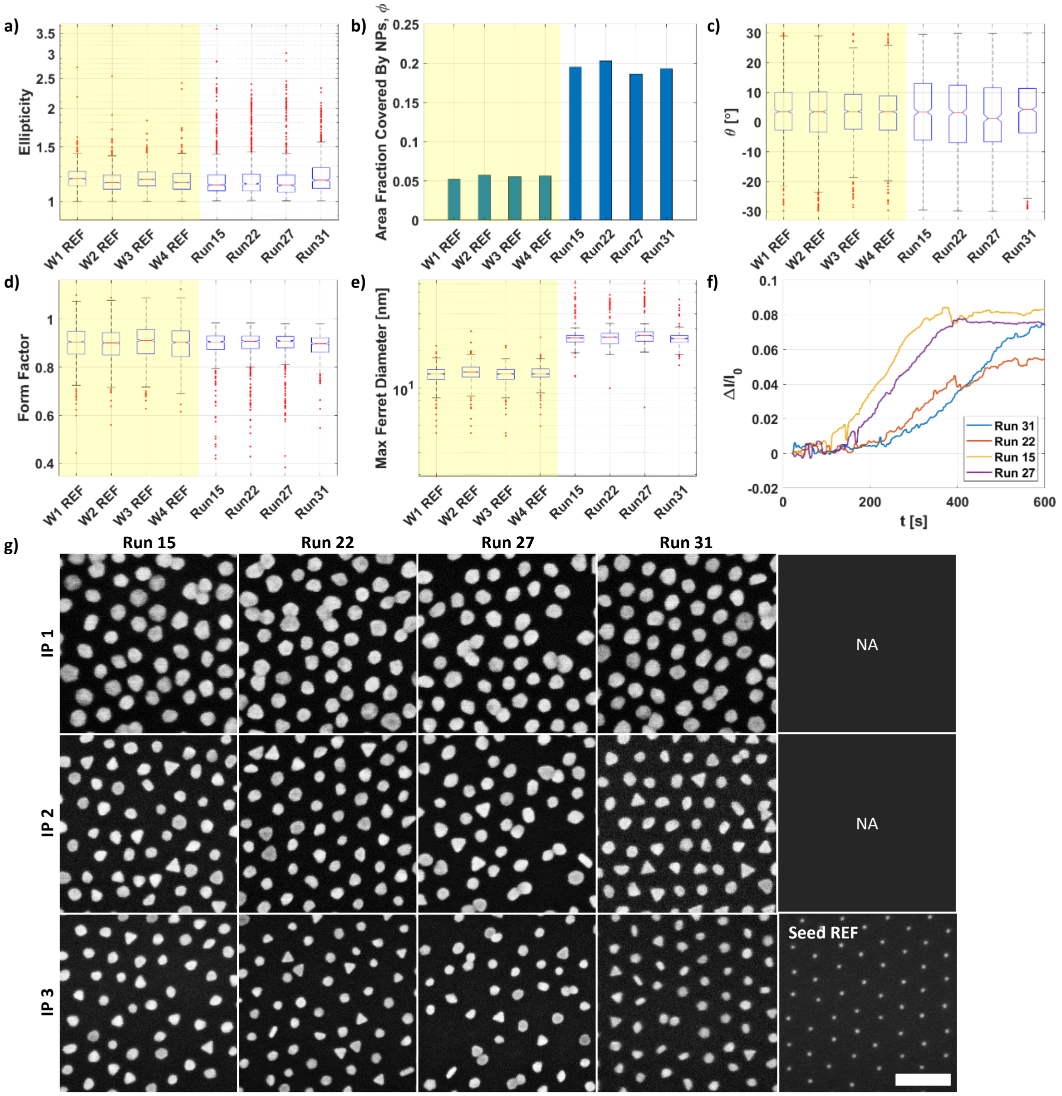

Figure S22: Compilation of data acquired from the DoE centerpoint replicates for both the samples and the unseeded media for the purpose of gauging the reproducibility of the SMNPG protocol. Panels a-e are plots of DIA-extracted features form SEM images acquired at IP2 on the DoE samples. Panel f is $\Delta I(t)/I_0$) of the corresponding unseeded media. Panel g) is a compilation of SEM images from the DoE centerpoint replicate samples at all three inspection points (IP1-3). NB: The sample run here labeled "31" is actually sample DoE run 1. The scale bar equals 100 nm.

Table S5: Summary of DIA-extracted Data After SMNPG with No Growth Medium Agitation

m [g]	$(m - \langle m \rangle)/\langle m \rangle$ [%]	$c_{citrate}$ [µM]	c_{HAuCl4} [µM]	c_{H2O2} [M]	$\Delta N/N_0$	φ	$\bar{X}_{Area}$ [nm^2]	s_{Area} [nm^2]	$\bar{X}_{Max\ FD}$ [nm]	$s_{Max\ FD}$ [nm]	$\bar{X}_{FF}$	s_{FF}	$\bar{X}_{Ellip.}$	$s_{Ellip.}$	s_d [nm]	s_θ [deg.]
[a]-	-	-	-	-	0.00	0.025	59	14	8.8	1.1	0.933	0.044	1.10	0.07	2.9	10.9
0.3978	1.8	340	100	5.06	-0.03	0.085	191	24	16.3	1.2	0.911	0.022	1.12	0.08	2.9	10.4
0.3836	-1.8	"	"	1.26	-0.01	0.087	186	31	16.1	1.5	0.907	0.027	1.13	0.11	3.2	10.4
0.3841	-1.7	"	"	0.25	-0.03	0.117	250	42	19.5	2.2	0.891	0.033	1.23	0.17	3.6	10.4
0.3976	1.7	"	"	0.025	-0.06	0.154	333	64	22.9	3.2	0.875	0.050	1.28	0.30	3.7	11.3

Comment: The DIA was perfomed on SEM-images aquired at Vacc = 5 kV and 400k magnification. m is the mass of the sample. $\Delta N/N_0$ ***and*** φ are the relative change in counted NPs and the fractional area coverage of NPs respectively. $\bar{X}_{Area}$, $\bar{X}_{Max\ FD}$, $\bar{X}_{FF}$, $\bar{X}_{Ellips.}$ and $\bar{X}_d$ are the mean NP cross-sectional area, max Ferret diameter, fill factor and center-to-center interparticle distance respectively while s_{Area}, $s_{FD\ max}$, s_{FF}, $s_{Ellips.}$ and s_d are the corresponding standard deviations. s_θ is the standard deviation of the discrepancy between the angle spanned by the centroids of the two nearest neighbours and that of a perfect hexagonal geometry. t_{SMNPG} = 10 min. [a]Seed reference.

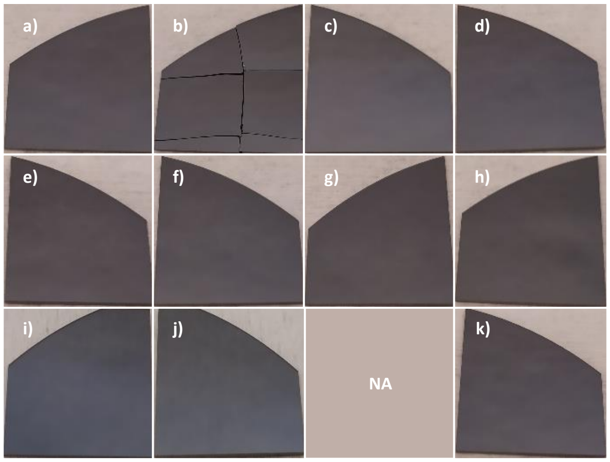

Figure S23: Compilation of samples photographed after the SMNPG procedure to visually gauge the deposition uniformity. Panels a-d constitute a sample series with progressively decreasing c_{H2O2} during SMNPG with no growth medium agitation. c_{H2O2} was a) 5.06 M, b) 1.26 M, c) 0.25 M and d) 0.025 M (Table S5). Panels e-h constitutes a series with progressively increased amount of PVA added to the growth medium (Table S6 entries 2-5). Panels m and n display control samples included in the PVA experiment (Table S6 entries 6 and 7) while panel o is a seed reference sample.

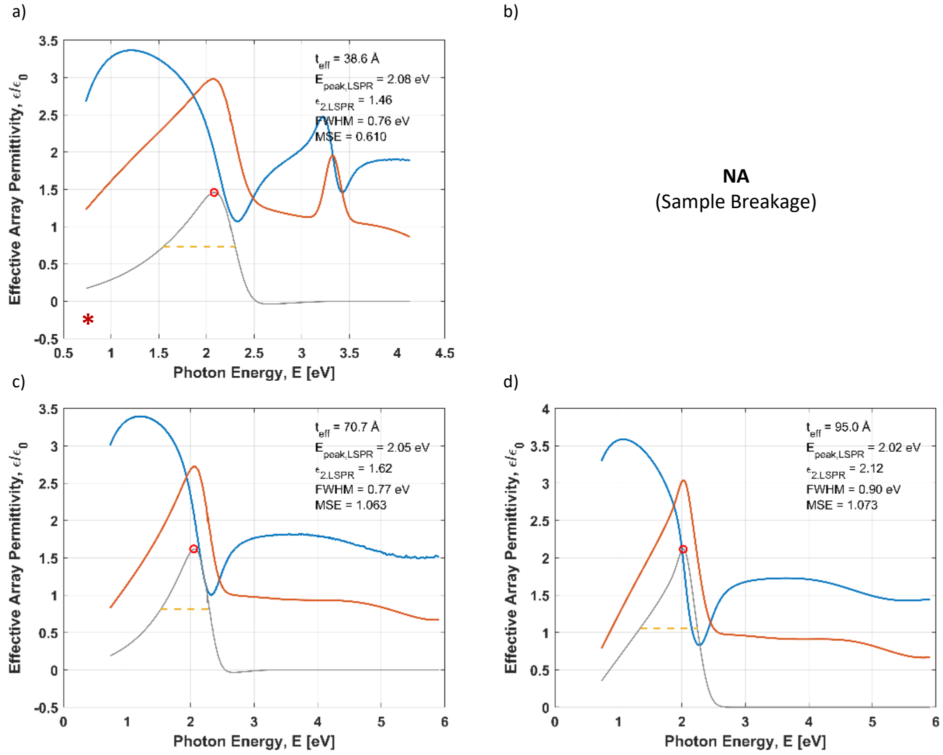

*Figure S24: Permittivity (ϵ) modelled based on SE data acquired on samples for which no agitation was used during the SMNPG. $c_{HAuCl4} = 100\ \mu M$ and $c_{citrate} = 340\ \mu M$ were consistently used for all samples whereas c_{H2O2} was progressively decreased: a) 5.06 M, 1.26 M, 0.25 M and d) 0.025 M. *Sample was measured at an incidence angle (α) of 61°, as opposed to the rest for which $\alpha = \{65.70, 75\}°$ – note the two additional (artifact) peaks at high E.*

PART II

Use of Poly(vinyl alcohol) Instead of, or in Conjunction with, Citrate During SMNPG

As higher $c_{citrate}$ was previously observed to be affiliated with more extensive *homogenous nucleation* (HN) in solution, alternative complexing and capping agents are of interest. In a second follow-up experiment we thus investigated the effect of using *poly(vinyl alcohol)* (PVA) instead of, or in conjunction with, citrate in our reaction system. PVA is an inexpensive, non-toxic and widely available polymeric capping agent. The experiment consisted of two parts; in the first, unseeded growth media were prepared analogously with those in the corresponding DoE scheme except that PVA was added either instead of or after the citrate from a 1% (w/w) aq. stock solution. Total medium volume (40 mL) was maintained. In the second part, SMNPG was performed, using no growth medium agitation and an extended *sample immersion time* ($t_{SMNPG} = 10$ min), on seed-decorated samples.

In running the unseeded media, we found that HN was not an issue (as inferred from $\Delta I(t)/I_0$) when replacing 400 µL 1% (w/w) citrate(aq) ($c_{citrate} = 340\ \mu M$ in medium) with an equal volume of 1% (w/w) PVA(aq). Even more interesting is the seeming ability to tune the onset of the $\Delta I(t)/I_0$ increase with the concentration of PVA, when added to a SMNPG medium containing with the highest DoE concentration levels of citrate and H_2O_2 (Figure S25 a). Close to complete suppression of the $\Delta I(t)/I_0$ change can be achieved at lower c_{H2O2} over the span of ≥15 min (Figure S25 b).

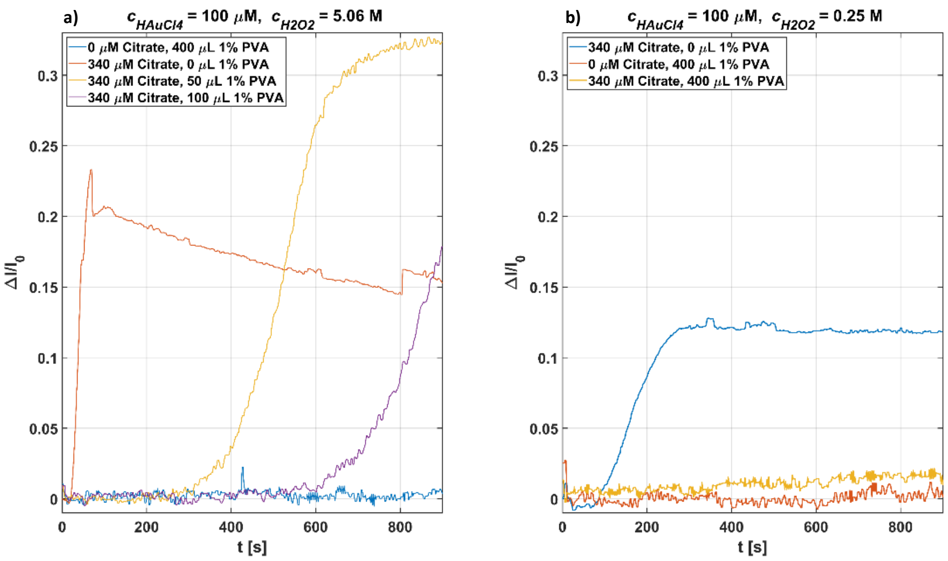

Figure S25: $\Delta I(t)/I_0$ when using PVA instead of, or in conjunction with, citrate in unseeded growth media. $c_{HAuCl4} = 100\ \mu M$ was used in all media whereas c_{H2O2} was 5.06 M and 0.25 M for those in a) and b) respectively. $c_{citrate}$ and the volume of 1% PVA stock solution ($V_{1\%\ PVA}$) in the individual runs are stated in the figure legends. In plotting the data, a 3-datapoint sweeping median filter was used for noise reduction.

The results from the unseeded media suggests that higher Au precursor utilization and improved reproducibility might be possible through the addition of PVA when conducting SMNPG on surface supported NPs. To investigate this possibility, we conducted SMNPG on a series of samples using no growth medium agitation, $c_{citrate} = 340$ µM, $c_{HAuCl4} = 100$ µM, $c_{H2O2} = 5.06$ M and a progressively increasing amount of added PVA. Two controls, for which no citrate and either no or 400 µL 1% PVA was used, were also included for reference (Figure S26 a).

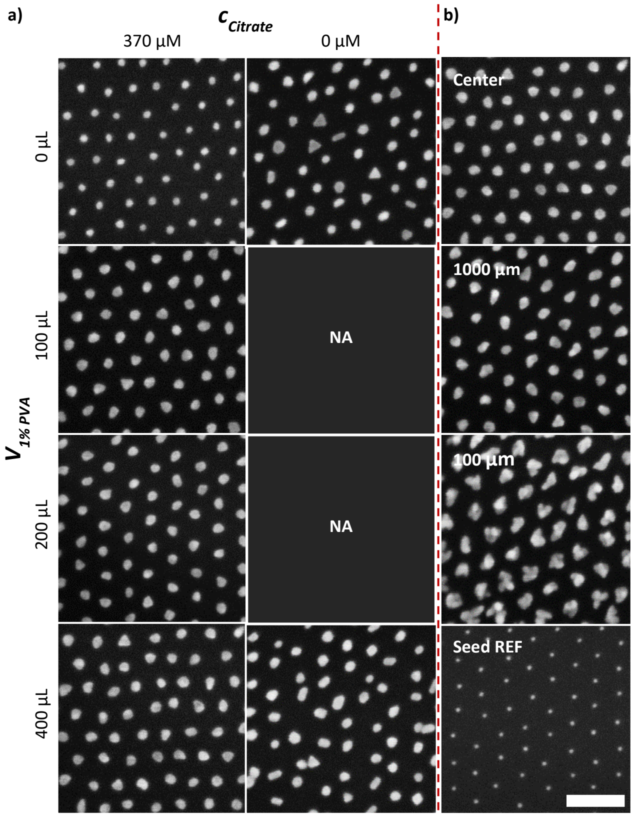

Figure S26: a) SEM images of surface supported NP arrays after performing SMNPG using poly(vinyl alcohol) (PVA) instead of, or in conjunction with, citrate during SMNPG. $c_{HAuCl4} = 100\ \mu M$ and $c_{H2O2} = 5.06\ M$ were consistently used for all samples whereas $c_{citrate}$ and $V_{1\%\ PVA}$ were varied as indicated in the figure. All images were acquired close to the sample center. b) Observation of how the NP shape and size change with distance from the sample edge when using a SMNPG recipe consisting of $c_{HAuCl4} = 100\ \mu M, c_{H2O2} = 5.06\ M, c_{citrate} = 340\ \mu M\ and\ V_{1\%\ PVA} = 400\ \mu L$. Location or distance from the edge stated in the panels. No medium agitation was used (in a and b) and the scale bar equals 100 nm.

Digital image analysis (DIA) of SEM images acquired at the sample centers, suggests that the size and ellipticity abruptly increase upon our lowest level of PVA addition and then remains fairly constant with increasing amount of PVA added. We also evaluated using only PVA or neither citrate nor PVA and obtained substantially higher ellipticity values in both cases than when using either citrate alone or the citrate-PVA combination (Table S6 and Figure S27). Moreover, more severe deterioration of the array pattern quality was seen when only using PVA (Figure S26 a). An intriguing phenomenon was observed when inspecting the sample for which citrate and the highest PVA concentration were used. The NPs close to the immediate sample edge exhibit a ‘popcorn-like’ morphology, although this appearance rapidly transitions to a more spherical one towards the sample center (Figure S26 b). This could potentially stem from a steric hindrance of adsorbed PVA chains, partially blocking reagent access to the NP surface.

Table S6: Summary of DIA Extracted Data After Using PVA Addition in the SMNPG Protocol

m [g]	*(m -<m>)/ <m>* [%]	t_{SMNPG} [min]	c_{HAuCl4} [uM]	$c_{Citrate}$ [µM]	V_{PVA} [µL]	c_{H2O2} [M]	*N*	*φ*	$\bar{X}_{Area}$ [nm²]	s_{Area} [nm²]	$\bar{X}_{Max FD}$ [nm]	$s_{Max FD}$ [nm]	$\bar{X}_{FF}$	s_{FF}	$\bar{X}_{Ellip.}$	$s_{Ellip.}$	s_d [nm]	s_θ [deg.]
[a]-	-	-	-	-	-	-	352	0.025	59	14	8.8	1.1	0.93	0.04	1.10	0.07	2.9	10.9
0.3959	6.7	10	100	340	0	5.06	339	0.071	162	24	15.0	1.2	0.91	0.02	1.12	0.09	3.3	9.3
0.3668	-1.2	"	"	340	100	"	326	0.129	293	35	21.0	1.7	0.89	0.03	1.20	0.15	2.8	9.4
0.3551	-4.3	"	"	340	200	"	332	0.126	273	37	20.1	1.8	0.89	0.02	1.19	0.12	2.6	9.0
0.3969	6.9	"	"	340	400	"	336	0.137	294	49	21.1	2.3	0.89	0.04	1.22	0.22	3.2	10.7
0.3619	-2.5	"	"	0	0	"	331	0.116	264	56	20.1	2.8	0.88	0.05	1.25	0.28	4.3	12.3
0.3504	-5.6	"	"	0	400	"	319	0.130	293	84	21.6	4.6	0.87	0.06	1.32	0.31	4.8	15.4

Comment: The DIA was perfomed on SEM-images aquired at Vacc = 5 kV and 400k magnification. ***m*** is the mass of the sample. ***ΔN/N₀ and φ*** are the relative change in counted NPs and the fractional area coverage of NPs respectively. $\bar{X}_{Area}$, $\bar{X}_{Max FD}$, $\bar{X}_{FF}$, $\bar{X}_{Ellips.}$ and $\bar{X}_d$ are the mean NP cross-sectional area, max Ferret diameter, fill factor and center-to-center interparticle distance respectively while s_{Area}, $s_{FD max}$, s_{FF}, $s_{Ellips.}$ and s_d are the corresponding standard deviations. s_θ is the standard deviation of the discrepancy between the angle spanned by the centroids of the two nearest neighbours and that of a perfect hexagonal geometry. [a]Seed reference.

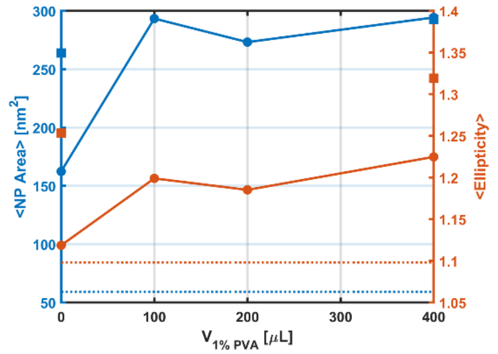

Figure S27: Mean area ($\bar{x}_{Area}$) and ellipticity ($\bar{x}_{Ellips}$) of surface supported NPs (at sample center) as functions of the amount of added PVA and citrate concentration. Circles and squares represent growth conditions involving $c_{citrate} = 340\ \mu M$ and $c_{citrate} = 0\ \mu M$, respectively. Note that edge pieces of slightly differing sizes were used (Table S6).

All of the samples included in this experiment appear uniform upon visual inspection (Figure S25 i-o) and, as with the DoE samples, optical characterization using *spectroscopic ellipsometry* (SE) was performed (Figure S28).

To summarize, the use of the combination of PVA and citrate seems like a viable option for increasing the gold precursor utilization. That might, however, come at the cost of a more jagged NP shape in cases of more extensive growth. Looking forward, further optimization of the PVA concentration and/or molecular weight might prove useful in addressing this issue but alternative capping/complexing agents are likely of more interest. One candidate in the latter scenario might be tris-base as used by Li et al.[28]

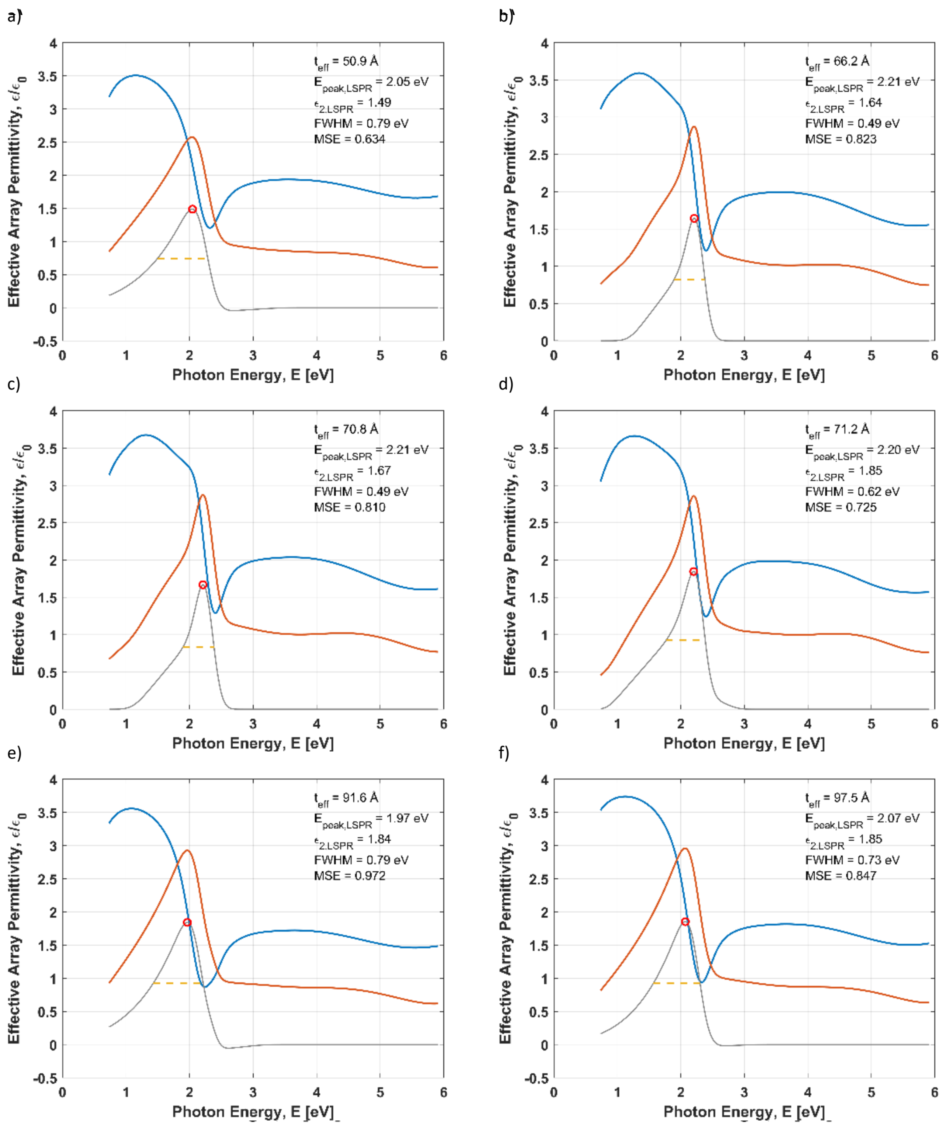

Figure S28: Effective thickness (t_{eff}) and permittivity (ϵ), modelled based on acquired SE data, of surface supported NP arrays on samples in a series investigating the effects of PVA addition during SMNPG. $c_{HAuCl4} = 100\ \mu M$ and $c_{H2O2} = 5.06\ M$ were consistently used for all samples whereas $c_{citrate}$ and $V_{PVA\,1\%}$ varied accordingly: a) {$c_{citrate} = 400\ \mu M$, $V_{PVA\,1\%} = 0\ \mu L$}, b) {$c_{citrate} = 400\ \mu M$, $V_{PVA\,1\%} = 100\ \mu L$}, c) {$c_{citrate} = 400\ \mu M$, $V_{PVA\,1\%} = 200\ \mu L$}, d) {$c_{citrate} = 400\ \mu M$, $V_{PVA\,1\%} = 400\ \mu L$}, e) {$c_{citrate} = 0\ \mu M$, $V_{PVA\,1\%} = 0\ \mu L$}, f) {$c_{citrate} = 0\ \mu M$, $V_{PVA\,1\%} = 400\ \mu L$}. Line color legend: blue $= \epsilon_1(\lambda)$, orange = $\epsilon_2(\lambda)$, gray = ϵ_2 contribution of oscillators corresponding to the LSPR peak, dashed line $= FWHM$.
